# Inhibitory feedback control of NF-κB signalling in health and disease

**DOI:** 10.1042/BCJ20210139

**Published:** 2021-07-16

**Authors:** Jack A. Prescott, Jennifer P. Mitchell, Simon J. Cook

**Affiliations:** Signalling Programme, The Babraham Institute, Babraham Research Campus, Cambridge CB22 3AT, U.K.

**Keywords:** decoy receptors, deubiquitylases, feedback, IκBs, IKKs, nuclear factor κB

## Abstract

Cells must adapt to changes in their environment to maintain cell, tissue and organismal integrity in the face of mechanical, chemical or microbiological stress. Nuclear factor-κB (NF-κB) is one of the most important transcription factors that controls inducible gene expression as cells attempt to restore homeostasis. It plays critical roles in the immune system, from acute inflammation to the development of secondary lymphoid organs, and also has roles in cell survival, proliferation and differentiation. Given its role in such critical processes, NF-κB signalling must be subject to strict spatiotemporal control to ensure measured and context-specific cellular responses. Indeed, deregulation of NF-κB signalling can result in debilitating and even lethal inflammation and also underpins some forms of cancer. In this review, we describe the homeostatic feedback mechanisms that limit and ‘re-set’ inducible activation of NF-κB. We first describe the key components of the signalling pathways leading to activation of NF-κB, including the prominent role of protein phosphorylation and protein ubiquitylation, before briefly introducing the key features of feedback control mechanisms. We then describe the array of negative feedback loops targeting different components of the NF-κB signalling cascade including controls at the receptor level, post-receptor signalosome complexes, direct regulation of the critical ‘inhibitor of κB kinases’ (IKKs) and inhibitory feedforward regulation of NF-κB-dependent transcriptional responses. We also review post-transcriptional feedback controls affecting RNA stability and translation. Finally, we describe the deregulation of these feedback controls in human disease and consider how feedback may be a challenge to the efficacy of inhibitors.

## Background — The NF-κB transcription factors

**Figure 1.**
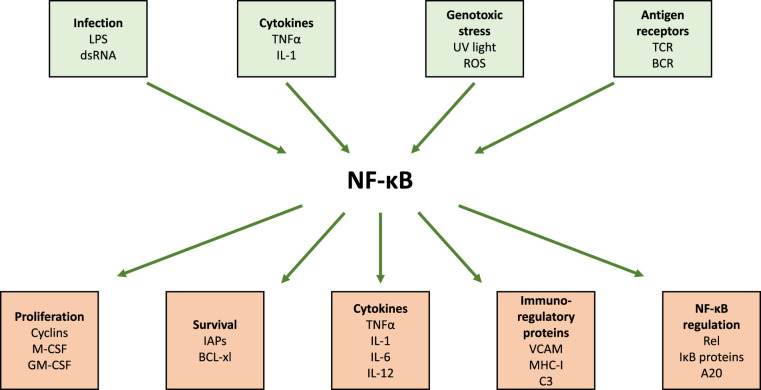
Input and output of NF-κB activity. Activation of NF-κB occurs as a consequence of a vast array of stimuli including infectious pathogens, cytokines, environmental and chemical stresses, hormones and others. Once activated, NF-κB controls many transcriptional outcomes, driving the expression of proteins involved in immune regulation, cell survival, cell-cycle progression, as well as regulators of NF-κB itself creating a negative feedback loop.

The ability of cells to adapt to changes in their environment is essential if they are to maintain their physiological integrity in the face of mechanical, chemical or microbiological stress. The transcription factor, nuclear factor-κB (NF-κB), is a critical component that controls inducible gene expression as cells attempt to restore homeostasis. It plays a key role in the response to infection, both in immune and non-immune cells, but it also has roles in cell survival, differentiation and proliferation, controling the expression of hundreds of biologically important genes including regulators of apoptosis, stress-response genes, cytokines, chemokines, growth factors and their receptors (Figure 1). It also has a role in development where its activation is essential for the formation of secondary lymphoid organs [1]. NF-κB is activated by a vast array of stimuli or cues and is regulated by a multitude of elements at different stages throughout the signalling pathway. Failure to regulate NF-κB can result in devastating consequences for the host, such as chronic inflammatory disease, cardiovascular disease, neurodegenerative disorders and cancer [2].

**Figure 2.**
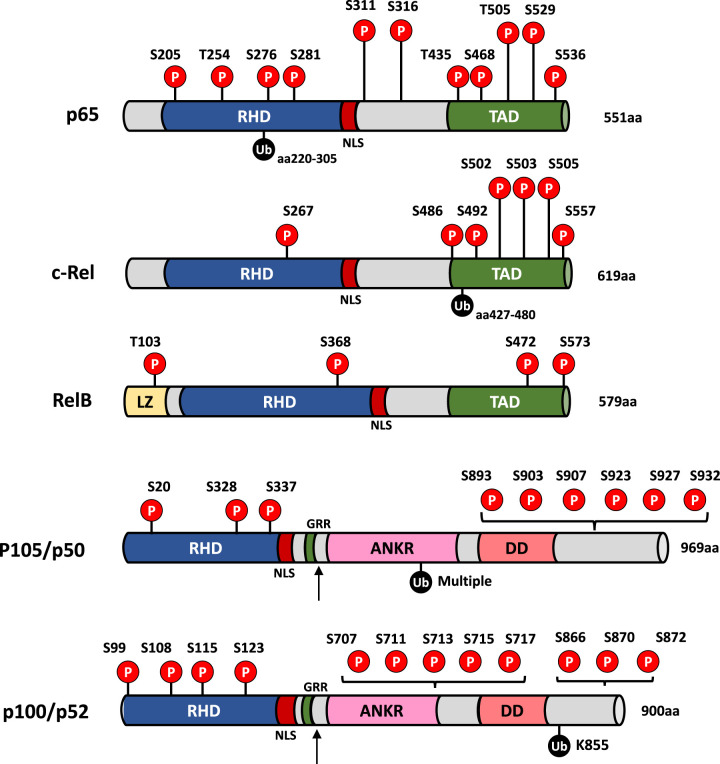
Domain organisation of the NF-κB family of transcription factors. The NF-κB proteins are a family of five related transcription factors; p65, c-Rel, RelB, p50 and p52, the latter two being derived from the limited proteasomal processing of precursors p105 and p100, respectively. Each of the members contains a conserved 300 amino acid long N-terminal Rel homology domain (RHD) that mediates DNA binding, dimerisation, IκBα interaction and nuclear translocation (NLS). The family can be divided into two groups based on their transactivation potential; p65, RelB and c-Rel contain a C-terminal transactivation domain (TAD) that is responsible for the transcriptional activity of dimers containing these subunits. p50 and p52 lack a TAD, and thus act as transcriptional repressors in their homodimeric form. p100 and p105 are also classified as IκB proteins due to their C-terminal ankyrin repeats (ANKR), which enable them to bind to other NF-κB subunits and inhibit their nuclear localisation. A glycine rich region (GRR) in p105 and p100 prevents complete proteolysis to generate p50 and p52. An arrow indicates the approximate location of the C-terminal residues of p50 and p52 generated. The C-terminal death domains (DD) of p100 and p105 mediate protein interactions with adaptor proteins involved in regulating apoptosis, NF-κB and AP-1 pathways. In p105 it also mediates interactions with IKK kinases, while in p100 it functions as a processing inhibitory domain (PID) that restricts basal processing to p52. RelB is unique in that it also contains an N-terminal leucine zipper (LZ) region that is required alongside its TAD to be fully active. RelB cannot form homodimers, and preferentially binds to p100/p52. Phosphorylations that have been shown to regulate NF-κB are highlighted on each subunit. Numbering corresponds to human amino acid sequence. Ub refers to K48 linked polyubiquitylation.

**Figure 3.**
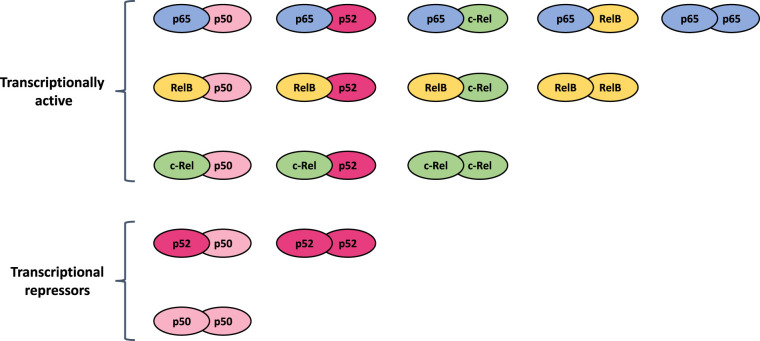
Dimer configurations of NF-κB subunits. The presence of the Rel homology domain allows the NF-κB subunits to form either homo- or heterodimers. The presence of the transactivation domain in p65, RelB and c-Rel confers transcriptional activity on dimers that contain these subunits, whereas p50 and p52 lack this domain and so lack transcriptional activity. *In vivo*, RelB has only been observed to form dimers with p50 and p52 so only 12 dimer configurations are thought to exist in cells.

NF-κB in mammals is a family of five related proteins (Figure 2) that form dimers capable of positively or negatively regulating gene expression. These subunits are p65 (also known as RelA), RelB, c-Rel as well as p105 and p100, which undergo further proteolytic processing into the active subunits p50 and p52, respectively. The subunits share an evolutionarily conserved 300 amino acid ‘rel homology domain’ (RHD) which enables dimerisation and binding to κB sites, regulatory elements found in NF-κB target genes. Furthermore, p65, RelB and c-Rel contain a transcriptional transactivation domain (TAD) at their C-terminus which confers the ability to promote gene expression. p50 and p52 lack a TAD and when present as homodimers are thought to act as transcriptional repressors, competing with transcriptionally active dimers to bind to their DNA targets. However, p50 and p52 can promote transcription when they are bound to p65, RelB or c-Rel as part of a heterodimer, giving them a dual role. Theoretically there are 15 possible combinations of dimers (Figure 3) but RelB has only been observed to form dimers with p50 and p52 so only 12 dimers are known to exist in cells.

**Figure 4.**
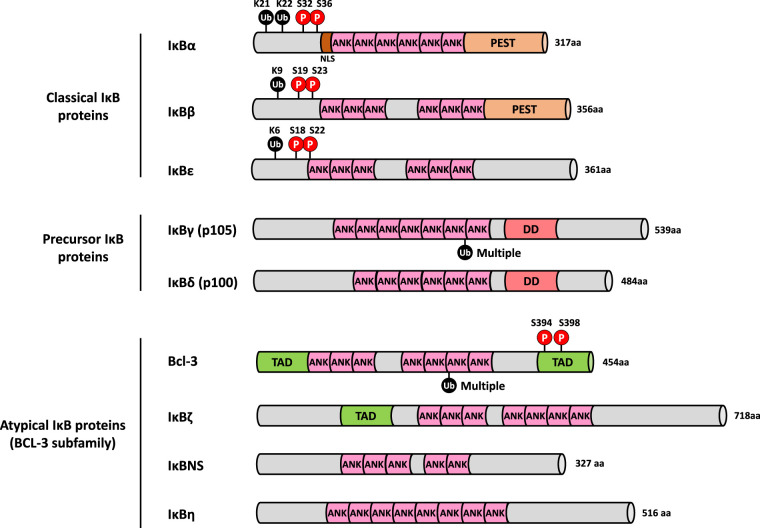
Domain organisation of the IκB family proteins. The nine bona fide IκB family proteins contain numerous ankyrin repeats, which formally defines the family. The ‘classical’ IκB proteins are: the prototypical member, IκBα; IκBβ; and IκBε. They function by sequestering NF-κB dimers in the cytosol in unstimulated cells, and are degraded in an IKK-dependent manner upon pathway activation. The sites of IKK-dependent phosphorylation and β-TrCP-dependent K48-linked polyubiquitylation are shown. The ‘precursor’ IκB proteins, p105 and p100, act like the classical IκB proteins to sequester NF-κB subunits in the cytosol in the unstimulated state. Proteasomal-dependent limited proteolysis of p105 and p100, however, results in the liberation of p50 and p52 NF-κB subunits, respectively. In the case of p100 this is typically induced in a ubiquitin-dependent manner downstream of activation of the non-canonical NF-κB pathway, while p105 processing is thought to occur largely constitutively, both co- and post-translationally, and in a ubiquitin-independent manner. The ‘atypical’ IκB proteins include BCL-3, IκBζ, IkBNS and IκBη. Their expression is typically low and is induced by various stimuli, including NF-κB activation. Unlike the other IκB family members, they localise to the nucleus where they bind to DNA-associated NF-κB dimers to exert both positive and negative effects on NF-κB-mediated transcription. Ub, ubiquitin. P, phosphorylation site. ANK, ankyrin domain, PEST, sequence motif rich in proline (P), glutamate (E), serine (S) and threonine (T). DD, death domain. TAD, transactivation domain.

**Figure 5.**
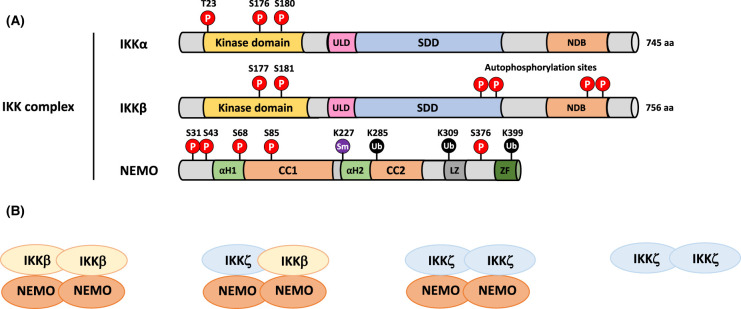
Domain organisation of the members of the IKK complex. (**A**) Schematic of IKK proteins. Both IKKα and IKKβ have a kinase domain at the N-terminus. Phosphorylation of serine residues in this activation loop of this domain is essential for their kinase activity. IKKβ has a number of serine residues towards its C terminus that are autophosphorylated and that have the effect of decreasing IKK activity after TNFα stimulation. IKKα and IKKβ also consist of a conserved ubiquitin-like domain (ULD), a scaffold/dimerisation domain (SDD) and a NEMO-binding domain (NBD) at the C-terminus. NEMO has two α-helical regions (αH1 and αH2), two coiled coil domains (CC1 and CC2), a leucine-zipper (LZ) and a zinc finger (ZF) domain. (**B**) Configuration of IKK dimers. The IKK proteins form either canonical complexes containing NEMO, or non-canonical, NEMO-independent IKKα homodimers. The canonical, NEMO-containing complexes may consist of homodimers of either IKKα or IKKβ, or a heterodimer of the two.

In resting cells, inactive NF-κB dimers are sequestered in the cytoplasm by members of a family of inhibitor of κB (IκB) proteins (Figure 4). These include IκBα, IκBβ, IκBε and the precursors p105 and p100 [3]; additionally, there are two atypical IκB proteins: B-cell lymphoma 3 (BCL-3) and IκBζ. They all share in common multiple ankyrin (ANK) repeat domains (ARDs). In the absence of an activating stimulus, IκBs bind and sequester NF-κB dimers in the cytoplasm, masking their nuclear localisation signal (NLS). Once an activating signal is received, the IκB proteins are rapidly phosphorylated by the IKKs [4]; this triggers IκB polyubiquitylation and proteasomal degradation, thereby liberating NF-κB dimers which translocate into the nucleus, bind to their DNA targets and regulate gene expression. These activation pathways are explored in greater detail in section 2. The crux of the NF-κB cascade is the IKK complex. It consists of two kinases: IKKα and IKKβ, plus a regulatory scaffold protein called NF-κB essential modulator (NEMO or IKKγ). There are two other related kinases, TBK1 and IKKε which share ∼30% sequence identity with the kinase domains of IKKα and IKKβ [5,6], but do not phosphorylate IκBα so will not be discussed further in this review. IKKα and IKKβ are serine/threonine kinases which share similar domain organisation (Figure 5). The scaffold/dimerisation domain (SDD) allows the IKKs to form stable homo- and heterodimers, and although the exact composition of the IKK complex *in vivo* remains the topic of great debate, the minimum stable composition of the canonical complex is thought to be an IKKα:IKKβ heterodimer bound to a NEMO dimer [7,8].

Aberrant regulation of NF-κB leads to the development of a number of pathologies [9]. Constitutive activation of NF-κB is detected in the synovium of rheumatoid arthritis (RA) patients [10], and increased activity is seen in various cell types in patients with inflammatory bowel disease (IBD) and ulcerative colitis (UC) [11]. However, the importance of inflammation in the subsequent development of cancer highlights NF-κB as a critical link between these pathologies. For instance, NF-κB is essential in the development of inflammation-driven, colitis-associated cancer (CAC) and hepatocellular carcinoma (HCC) [12,13].

Constitutive NF-κB activity in cancer cells can also promote tumour development in a cell-autonomous manner by promoting cell proliferation and preventing apoptosis by inducing the expression of anti-apoptotic genes such as the caspase inhibitor FLIP, the inhibitor of apoptosis proteins cIAP1/2 and XIAP, as well as pro-survival members of the BCL2 protein family [14]. NF-κB is constitutively active in cancer stem cells (CSCs), which are thought to mediate metastasis and resistance to therapy [15]. NF-κB stimulates CSC proliferation, survival, maintenance and expansion through the induction of SLUG, TWIST1 and SNAIL [16,17]. Furthermore, metabolomic changes are a recognised hallmark of cancer [18] and crosstalk between p53 and NF-κB regulates cancer cell metabolism. In cells with WT p53, NF-κB stimulates mitochondrial respiration by driving the expression of cytochrome c oxidase 2 (COX2); however, in p53-deficient cells, NF-κB supresses mitochondrial oxidative phosphorylation and promotes glycolysis by promoting the expression of genes that contribute to the Warburg effect [19,20].

This highlights the importance of stringent regulation of NF-κB to prevent the development of unwanted pathologies. Indeed, NF-κB signalling is subject to strict spatiotemporal control to ensure measured and context-specific cellular responses. In this review, we describe the homeostatic feedback mechanisms that limit and ‘re-set’ inducible activation of NF-κB. We first describe the key components of the signalling pathways leading to activation of NF-κB, including the prominent role of protein phosphorylation and protein ubiquitylation, before briefly introducing the key features of feedback control mechanisms. We then describe the array of negative feedback loops targeting different components of the NF-κB signalling cascade including controls at the receptor level, post-receptor signalosome complexes, direct regulation of the critical ‘inhibitor of κB kinases’ (IKKs) and inhibitory feedforward regulation of NF-κB-dependent transcriptional responses. We also review post-transcriptional feedback controls affecting RNA stability and translation. Finally, we describe the deregulation of these feedback controls in human disease and consider how feedback may be a challenge to the efficacy of inhibitors.

## Pathways of NF-κB activation

There are broadly two signalling pathways by which NF-κB is activated: the canonical, or classical pathway, and the non-canonical, or alternative pathway [2]; however, both culminate in the activation of the ‘inhibitor of κB kinase’ (IKK) complex. The canonical pathway mediates the rapid and reversible inflammatory response. Conversely, the ‘developmental response’ is slower, irreversible and mediated via the non-canonical pathway. The former is dependent upon the regulatory NF-κB essential modulator (NEMO) component of the IKK complex whereas the latter is independent of it [21]. Each pathway is described below including evidence of some crosstalk between them.

### The canonical pathway

**Figure 6.**
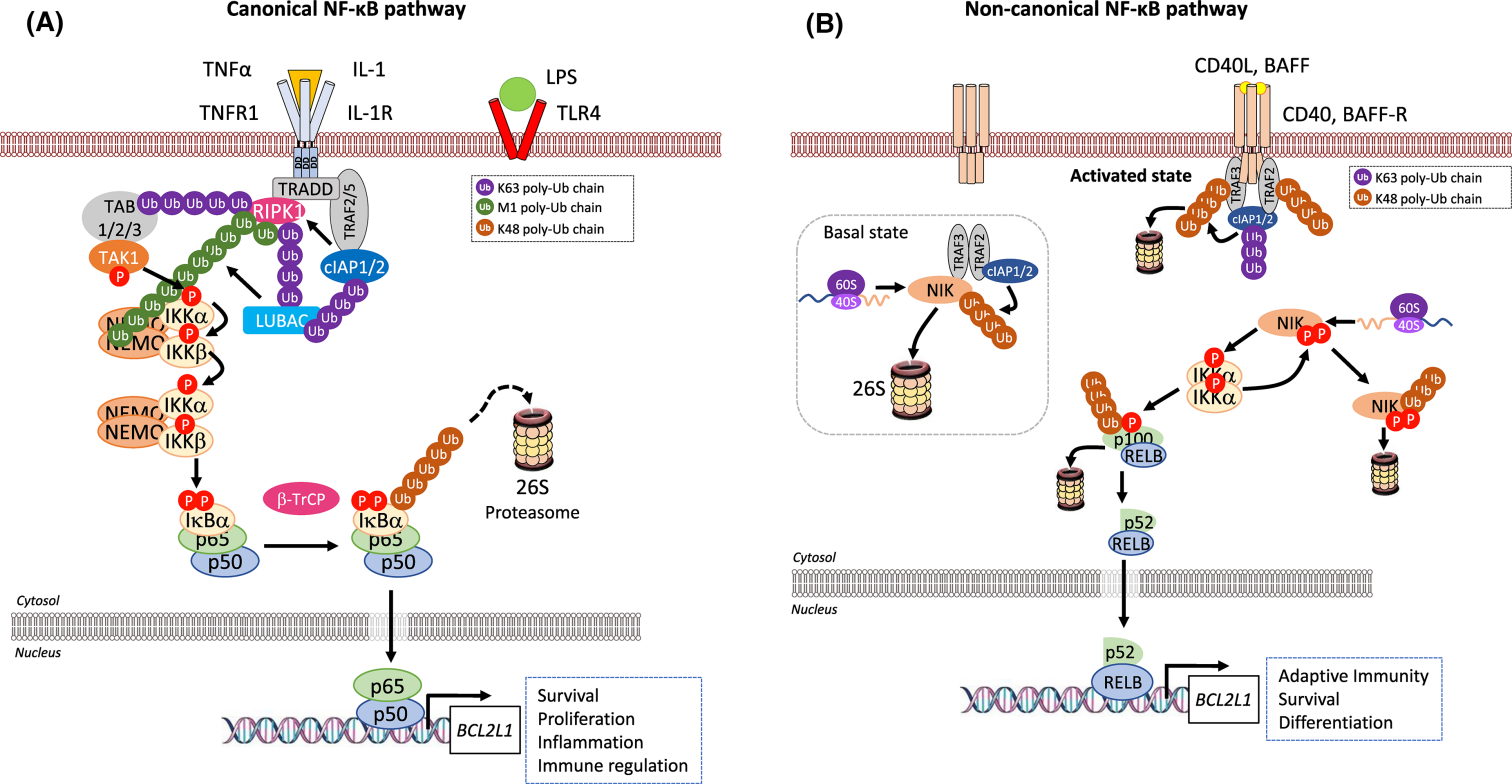
Overview of the canonical and non-canonical NF-κB signalling pathways. (**A**) The canonical pathway. The mechanism of activation of canonical NF-κB signalling pathway is best characterised for inflammatory cytokines, such as IL-1 or, as described here, TNFα. TNFα binding to the extracellular domain of the receptor leads to translocation of TNFR1 to lipid rafts and recruitment of TRADD to the cytoplasmic death domains of TNFR1. TRADD, in turn, recruits RIPK1 kinase (RIPK1), and subsequently the TRAF2 or TRAF5 adaptor proteins and cIAP1 or cIAP2 to assemble TNFR1 complex I. cIAP1 and cIAP2 generate K63-linked polyubiquitin chains on RIPK1 and other components of the complex. This is necessary to recruit LUBAC, which stabilises complex I by catalysing the attachment of linear M1-linked polyubiquitin chains, typically to RIPK1. K63-polyubiquitylated RIPK1 also recruits the TAK1:TAB complex. LUBAC-mediated M1-linked linear polyubiquitylation of RIPK1 then promotes the recruitment of NEMO the critical regulatory scaffold of the IKK complex. Binding of NEMO to ubiquitin chains is thought to induce a conformational change that contributes to the activation of associated IKK kinases. Membrane proximal recruitment of IKK kinases also contributes to IKK activation through proximity to TAK1, which is thought to prime the activation of IKK via phosphorylation of S176/S177 of IKKα/IKKβ, and through oligomerisation of IKK complexes, which is thought to facilitate trans-autophosphorylation of the T loop, leading to full activation. The active IKK complex subsequently phosphorylates IκB proteins at S32 and S36 (in IκBα) and this phosphodegron recruits β-TrCP, which is part of the E3 ubiquitin ligase SCF^β-TrCP^ (S phase kinase-associated protein 1 (SKP1)-cullin 1-F-box protein containing β-transducin repeat-containing protein). SCF^β-TrCP^ promotes the K48-linked ubiquitylation and proteasomal degradation of IκBα. This enables NF-κB complexes, such as p65-p50, to accumulate in the nucleus, where they activate NF-κB target genes involved in survival, proliferation, inflammation and immune regulation. (**B**) The non-canonical pathway. In the basal state (shown in the box), a TRAF2/3-cIAP1/2 complex catalyses the constitutive degradation of the protein kinase NIK. TRAF3 recruits NIK to the complex via dimerisation with TRAF2. cIAP1/2, recruited to the complex by interaction with TRAF2, catalyses the K48-linked polyubiquitylation of NIK, priming it for proteasomal degradation. This blocks the accumulation of NIK and hence prevents activation of non-canonical NF-κB signalling. Activation of the non-canonical pathway is driven by binding of ligand (e.g. CD40L, BAFF) to a subset of the TNF receptor superfamily members such as CD40 and BAFF-R. Ligand engagement induces the recruitment of TRAF2, TRAF3 and cIAP1/2 to the receptor. Here the K48-specific E3 ligase activity of cIAP1/2 switches from NIK to TRAF3, resulting in TRAF3 degradation. In some cases, TRAF2 is also targeted for degradation. In the absence of TRAF3, *de novo* synthesised NIK protein is stabilised, accumulates and phosphorylates IKKα at S176 to activate its kinase activity. NIK also recruits p100 to IKKα leading to IKKα phosphorylating p100. Phosphorylated p100 undergoes K48-linked ubiquitylation by β-TrCP and is targeted for limited proteolysis. A glycine rich region in p100 prevents complete proteasomal degradation. This releases the NF-κB subunit p52 and enables it to productively dimerise with RelB and translocate into the nucleus to regulate target gene expression. Notably, NIK-dependent activation of IKKα is proposed to be independent of NEMO.

The canonical pathway is activated by a variety of stimuli, including pro-inflammatory cytokines, pattern-associated molecular patterns (PAMPs) or damage-associated molecular patterns (DAMPs) binding to cognate receptors. This triggers a signalling cascade culminating in the activation of the IKK complex through phosphorylation of serines (S177 and S181 of IKKβ) in their kinase activation loop. The IKKβ subunit is thought to be the predominant component contributing to canonical NF-κB activation because IKKβ (*Ikbkb*) and p65 (*Rela*) knock out (KO) mice show a similar phenotype [22,23]. It exists in a complex with IKKα and NEMO. The signalling complex that coordinates activation of the IKK complex is referred to as the signalosome complex (SC) and is specific to the receptor, and hence ligand, stimulating the pathway. Canonical signalling is exemplified by the response to tumour necrosis factor α (TNFα) (Figure 6A) where, upon binding of TNFα to the TNF receptor (TNFR), TRADD and RIPK1 are recruited to the receptor via its death domain. TRAF2 is recruited via TRADD which itself recruits the E3 ligases cIAP1 and 2, which polyubiquitylate RIPK1 [24]. These ubiquitin chains act as anchors binding TAB/TAK1, allowing TAK1 to phosphorylate and activate the IKKs. Furthermore, the formation of linear methionine 1 (M1) ubiquitin chains can also activate the IKK complex when the E3 ligase LUBAC is recruited by TRADD, TRAF2 and cIAP1/2 and then ubiquitylates NEMO. This is thought to activate IKKs by promoting *trans* autophosphorylation [25]. The canonical pathway is also activated by other ligands including interleukin-1 (IL-1) as recognised by the IL-1 receptor (IL-1R), the gram-negative bacterial component lipopolysaccharide (LPS) as recognised by Toll-like receptor 4 (TLR4), and T-cells as recognised by the T-cell receptor (TCR). These lead to different signalling components being recruited. In the case of IL-1R and TLRs, binding of their cognate ligands allows their intracellular Toll Il-1 receptor (TIR) domains to recruit TIR-containing adapter proteins such as MyD88, TRIM, Mal or TRAM, depending on the specific ligand and receptor [26]. Dimerised MyD88 recruits IRAK4 which binds IRAK1 and this leads to the recruitment of the E3 ligase TRAF6. TRAF6 conjugates K63-linked polyubiquitin chains onto IRAK1. This facilitates the binding and phosphorylation of IKKs by TAK1 [27]. Upon activation, the IKKs phosphorylate the classical IκB proteins (at S32/S36 on IκBα), triggering their K48-linked polyubiquitylation by the SCF^βTrCP^ E3 ligase complex, marking them for degradation by the 26S proteasome [28]. Canonical signalling liberates predominantly p65- and c-Rel-containing NF-κB dimers which enter the nucleus to bind to their DNA targets and promote gene expression.

### The non-canonical pathway

The non-canonical NF-κB pathway is activated by the wider TNF superfamily receptors (TNFSFR) [29] including CD40, lymphotoxin β receptor (LTβ-R), B-cell activating factor receptor (BAFF-R) and receptor activation of NF-κB (RANK) [30,31]. Non-canonical NF-κB activation is mediated through an IKK complex that is proposed to consist of an IKKα homodimer without NEMO, and is dependent upon the cellular accumulation of NF-κB-inducing kinase (NIK) (Figure 6B). In the absence of a stimulus TRAF3 and cIAP1/2 bind to NIK and target it for K48 polyubiquitylation and proteasomal degradation. Upon receipt of an activating signal, TRAF2 recruits and K63-polyubiquitylates cIAP1/2. TRAF3 acts as an adaptor molecule that facilitates the formation of a complex composed of NIK, TRAF2 and cIAP1/2 which K48-polyubiquitylates TRAF3 causing it to be degraded by the proteasome [32]. Since TRAF3 normally represses NIK levels this leads to the accumulation of NIK and activating phosphorylation of IKKα at S866/870 which activates it; IKKα then phosphorylates the NF-κB precursor protein, p100. This acts as a signal causing p100 to be recognised and polyubiquitylated by SCF^βTrCP^, marking it for the partial peroteolytic processing of its C-terminal ankyrin repeat domain (ARD). This releases its N terminus, now known as the p52 subunit of NF-κB, which dimerises with RelB, translocates to the nucleus and drives gene expression [33]. This processing event also eliminates the IκB property of p100. The C-terminus of p100 is structurally similar to other IκB proteins, containing multiple ARDs. It is not only able to sequester RelB:p52 NF-κB dimers in the cytoplasm, but also p65:p50 dimers which are traditionally considered effectors of canonical NF-κB signalling. Such cross-talk between the two activation pathways has led some to propose that NF-κB signalling should be viewed as a single system.

### Cross-talk between activation pathways

Almost all TNFSFRs which activate the non-canonical pathway can also activate the canonical pathway through their ability to stimulate the kinase activity of the canonical IKK complex via NIK-dependent phosphorylation of the IKKα subunit [34]; however this appears to be cell type-specific. Canonical p65:p50 dimers can be controlled by components of the non-canonical pathway. For example, mice that lack the form of p100 that can be processed were defective in p65-containing NF-κB dimers [35]. In *nik^−^*^/*−*^ osteoclast precursor cells, the accumulation of p100 leads to an enhanced association of p65 with p100 [36]. LTβ-R signalling during development causes the disruption of the IκBδ inhibitory complex, leading to the translocation of p65:p50 dimers to the nucleus [37]. This highlights IκBδ as a mediator of crosstalk between the canonical and non-canonical NF-κB pathways since it can inhibit p65 and is responsive to non-canonical developmental stimuli [21].

Transcriptional control of the non-canonical pathway by canonical NF-κB dimers is another level of cross-talk between the two pathways. Both *relb* and *nfkb2* (which encodes p100) genes contain κB binding sites in their regulatory regions and their transcriptional regulation is dependent upon p65 [38]. LTβ-R engagement can first activate canonical p65:p50 NF-κB dimers and then non-canonical RelB:p52 in mouse embryonic fibroblast (MEF) cells [39]; in addition, MEFs that lack p65 exhibit deficient activation of RelB-containing NF-κB dimers following LTβ-R stimulation, further indicating that p65 is required for the transcription of *relb* and *nfkb2*. Reconstituting RelB or IKKβ into these p65 KO MEFs restored LTβ-R-induced RelB activation, whereas p100 overexpression did not [40], which suggests that homeostatic *relb* expression as driven by p65 is the main determinant of non-canonical NF-κB signalling, rather than p65-induced p100 synthesis.

Finally, the overlap in functionality between NFKB1 (p105/p50) and NFKB2 (p100/p52) demonstrates the cross-talk between the two activation pathways. Cells from *nfkb2^−^*^/*−*^ mice that lack the RelB binding partner p52 form RelB:p50 dimers instead; these are not under the control of the canonical IκB proteins and so can compensate for the loss of non-canonical pathway-inducible RelB:p52 activity. Similarly, cells that lack *nfkb1* that encodes p50 form p65:p52 NF-κB dimers instead, with almost the same level of p65 activation and target inflammatory gene expression [41]. These and other studies highlight the complexity in understanding the conditions and consequences of NF-κB activation and downstream signalling.

## A brief introduction to feedback control

The term ‘negative feedback’ or ‘feedback inhibition’ refers to information control mechanisms in which the output from a pathway or system moderates the input so that it becomes self-regulating. This allows a pathway to be both responsive to an input or stimulus but also made stable or robust and able to adapt to interventions. Feedback controls in electrical amplification systems emerged in the early 20th century as did the concept of homeostasis in biological systems [42]. Parallels between feedback controls in machines and biological systems launched the field of ‘cybernetics’ to describe the science of self-regulating control systems in machines and living things [43].

Some of the earliest critical insights into feedback control in biology came from biochemists studying metabolic pathways in the bacterium *Eschericia coli*. In 1956 Edward Umbarger showed that isoleucine could inhibit the activity of threonine deaminase, the first enzyme in the pathway of isoleucine biosynthesis [44] whilst Arthur Pardee showed that aspartate transcarbamoylase, the first enzyme of the pyrimidine biosynthetic pathway, was inhibited by cytidine nucleotides, the end-products of the pathway (so-called ‘end-product inhibition’) [45]. By the late 1960s feedback inhibition of proximal enzymes in metabolic cascades by end-products acting at allosteric sites was widely accepted as a mechanism for matching supply of amino acids and nucleotides with demand for protein, RNA and DNA synthesis [46]. The ‘information’ carried in such feedback loops effectively ‘reports back’ the abundance of key metabolites, allowing the cell to marshall its resources so that they are produced only when required and also preventing products and intermediates from accumulating to levels that might otherwise be toxic.

Today biologists view feedback as a critical mechanism of homeostasis, allowing biological systems or their constituent pathways to adapt and operate within optimal parameters in the face of changes in their environment. Although the biochemical details and mechanisms are completely different, with prominent roles for protein–protein interactions and post-translational modifications (PTMs, such as protein phosphorylation or ubiquitylation), the ‘information’ carried in feedback loops in signalling pathways serves the same purpose as end-product inhibition in metabolic pathways. In signalling pathways, feedback loops report back on the activation of the pathway, thereby reducing further input or output; this provides tight controls on cellular responses to stimulation by hormones, growth factors and inflammatory cytokines. Since many signalling pathways control critical cell fate decisions, defects in such feedback loops can result in catastrophic pathway hyperactivation which can drive the development of acute and chronic disease.

**Figure 7.**
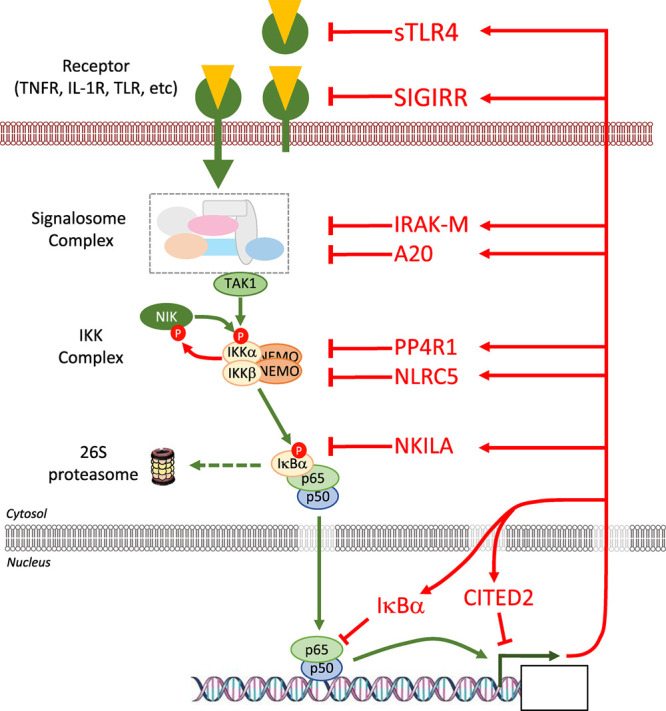
Feedback inhibition arising from NF-κB activation operates at all levels in the pathway. A simplified overview of the NF-κB signalling pathway in which a ligand-activated receptor (e.g. TNFR, IL-1R, TLR4) assembles and activates a signalosome complex which activates the ‘IKK kinases’ TAK1 or NIK. The activated IKKs phosphorylate IκBα to promote its proteasomal degradation, thereby releasing NF-κB (represented as a p65:p50 heterodimer) to enter nucleus and drive gene expression. Feedback regulation operates at virtually all levels of the pathway, from receptors to NF-κB gene transcription itself, as represented by the following examples. sTLR4, a soluble extracellular decoy receptor, is expressed following lipopolysaccharide stimulation and inhibits LPS-induced signalling by sequestration of LPS and/or blockade of the interaction between TLR4 and its co-receptors. SIGIRR is a transmembrane decoy receptor that lacks critical residues that are essential for signalling by IL-1R and so disrupts IL-1R receptor signalling. IRAK-M lacks kinase activity and disrupts IRAK-4-mediated phosphorylation of IRAK-1 and downstream IKK/NF-κB activation. A20 is a deubiquitylase that is expressed following NF-κB activation and removes K63 ubiquitin chains from multiple components to disrupt signalosome function; it also promotes K48-linked unbiquitylation and degradation of RIPK1; both effects block IKK activation and so prevent NF-κB activation. In the non-canonical pathway of NF-κB activation, stabilisation of NIK leads to phosphorylation and activation of IKKα to activate NF-κB; however, IKKα in turn phosphorylates the C-terminus of NIK to promote its proteasomal degradation as an inhibitory feedback mechanism to restore homeostasis. PP4R1, a regulatory subunit of the PP4c Ser–Thr phosphatase is expressed following activation of the T cell receptor and recruits PP4c to IKKα/β and catalyses their dephosphorylation and inactivation. NLRC5 expression is indirectly induced by LPS-dependent NF-κB activation via the NF-κB target gene, IFN-γ; NLRC5 binds to the IKKα/β kinase domains and blocks the binding of NEMO. NF-κB interacting long non-coding RNA (NKILA) is an LPS, IL-1β and TNFα-inducible NF-κB-regulated transcript that interacts with the NF-κB:IκBα complex in the cytoplasm and blocks IKK-dependent phosphorylation of IκBα. IκBα is itself an NF-κB target gene and de novo expressed IκBα can inhibit NF-κB activity by disrupting its interaction with DNA and by shifting the equilibrium of NF-κB subunit localisation from the nucleus to the cytoplasm. Finally, a variety of transcriptional repressors are recruited to DNA-bound NF-κB to inhibit gene transcription; an example is CITED2 which binds to the NF-κB coactivator p300, preventing p65 acetylation and inhibiting retention of p65 at target promoters. Further details on these exemplars of feedback regulation are provided in the text.

The signalling pathways leading to activation of NF-κB exemplify the complexity and importance of feedback controls. Feedback inhibition arising from pathway activation is critical for normal homeostasis and operates at all levels in the pathway (Figure 7). Here we use specific examples to describe the great diversity of these normal feedback controls in NF-κB signalling and describe the consequences when they are defective.

## Feedback at the receptor level

**Figure 8.**
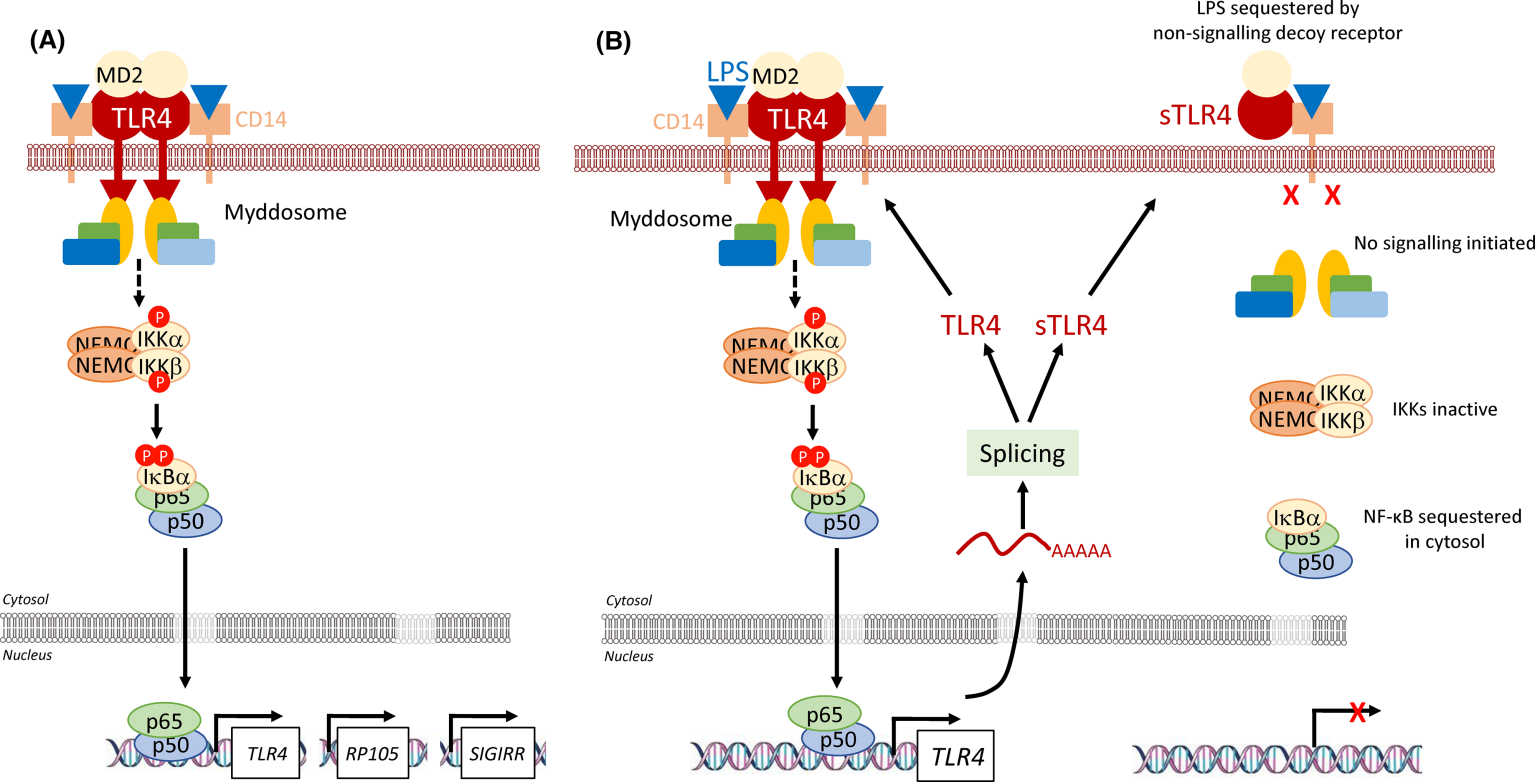

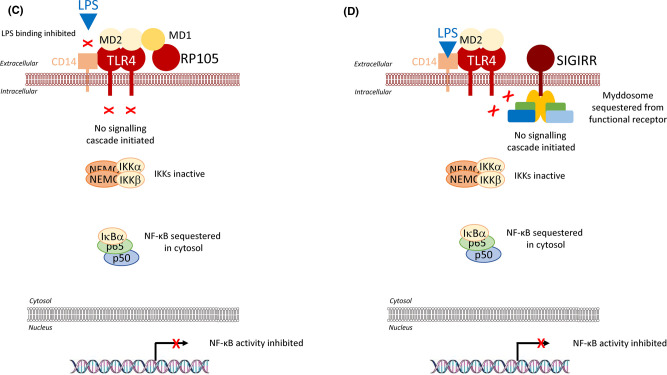
NF-κB dependent inhibitory feedback at the receptor level. (**A**) Activation of the NF-κB pathway downstream of TLR4 receptor. LPS is recognised by TLR4 and this leads to interaction with co-receptors CD14 and MD2. Following this stimulation, a number of proteins form a signalling complex collectively known as the ‘Myddosome’ which activates TAK1 to drive phosphorylation and activation the IKK complex. Following IκB phosphorylation and degradation NF-κB dimers enter the nucleus where they bind to κB sites in DNA and promote the transcription of target genes, including various ‘decoy receptors’ such as soluble TLR4, RP105 and SIGIRR. (**B**) Alternative splicing leads to the generation of soluble TLR4 (sTLR4) following NF-κB activation by LPS. This is secreted and sequesters LPS which is now not able to bind to full length TLR4. Consequently, all TLR4 signalling is disrupted and NF-κB activity declines. (**C**) RP105 and (**D**) SIGIRR are TLR4 homologs that are transcribed following NF-κB activation and serve as transmembrane decoy receptors. RP105 lacks an intracellular TIR domain and inhibits LPS-induced NF-κB activation by forming non-signalling complexes with TLR4 and MD2. SIGIRR contains one extracellular Ig domain and an intracellular TIR domain which lacks Ser447 and Tyr536 which are essential for IL-1R signalling. SIGIRR disrupts NF-κB activation by associating with IL-1R1, IRAK and TRAF6 in the Myddosome to disrupt these signalling complexes. See text for details.

Receptors that activate the NF-κB pathway are subject to negative feedback through the NF-κB-dependent induction of ‘decoy receptors’. Soluble decoys lack a transmembrane domain and sequester ligands outside the cell; transmembrane decoys bind ligands but lack intracellular domains and so are unable to relay signals to downstream targets. Soluble decoy receptors include sTLR4, a secreted splice variant of TLR4 that contains an extracellular domain [47]. sTLR4 expression is increased following lipopolysaccharide (LPS) stimulation (Figure 8A) and contributes to feedback inhibition of LPS-induced NF-κB activation, through sequestration of LPS and/or blockade of the interaction between TLR4 and its co-receptors, MD-2 and CD14 (Figure 8B). Another example is the soluble decoy receptor, serum stimulation-2 (sST2) [48,49]. LPS, IL-1 and TNFα stimulate the NF-κB-dependent expression and secretion of sST2 [50,51] which sequesters free IL-33. Examples of transmembrane decoy receptors include the TLR4 homolog, radioprotective 105 kDa protein (RP105) and single Ig IL-1 receptor (IL-R)-related molecule (SIGIRR). RP105 lacks an intracellular Toll/IL-1 receptor (TIR) domain [52], is expressed in response to LPS, and, in turn, inhibits LPS-induced NF-κB activation [53] by forming non-signalling complexes with TLR4-MD-2 (Figure 8C). SIGIRR contains only a single extracellular immunoglobulin (Ig) domain and has an intracellular TIR domain that lacks conserved residues (Ser447 and Tyr 536) that are known to be essential for signalling by IL-R1 [54]. SIGIRR is up-regulated in response to IL-1 and LPS [55] and associates with IL-1R1, IRAK and TRAF6 to disrupt IL-1R1/TLR4 receptor complexes [56] (Figure 8D). These examples show how NF-κB-dependent induction of decoy receptors links NF-κB pathway output to inhibition of the earliest upstream receptor signalling events.

## Inhibition of signalosome complex assembly

The signalosome complex that coordinates the activation of the IKKs is a major site of negative feedback regulation in NF-κB signalling pathways. The precise composition of the SC is specific to the ligand and receptor stimulating the pathway so many of the feedback mechanisms discussed here are highly signal-specific. Feedback regulation of the SC can be broadly categorised as: [1] expression of splice variants/homologous gene products that act in a dominant negative fashion to inhibit signal transduction upstream of IKK; [2] expression and/or recruitment of deubiquitylase enzymes (DUBs) that catalyse the removal of ubiquitin chains conjugated to components of the SC to promote its disassembly/inactivation; and [3] gene products that interfere with the assembly of the SC through inhibitory protein interactions.

### Dominant negative signal transducers

**Figure 9.**
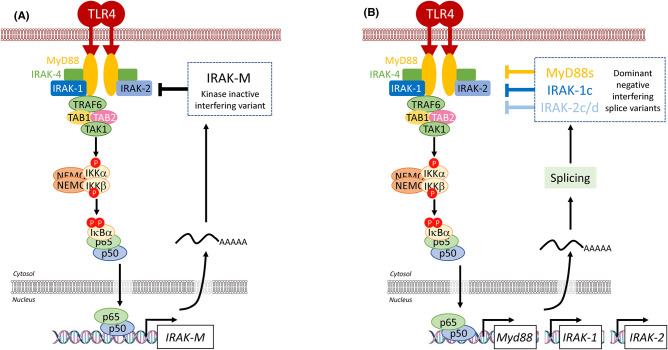
Inhibition of signalosome complex assembly by dominant negative signal transducers. (**A**) In human monocytes and macrophages, IRAK-M is induced by TLR ligands such as LPS. Lacking kinase activity, it acts as a negative regulator of IL-1R and TLR signalling by sequestering IRAK-1 and IRAK-4 in the signalling complex with MyD88 and so blocking phosphorylation of IRAK-1 by IRAK-4. Without this signal, IRAK-1 cannot associate with TRAF-6 and so downstream activation of the IKK complex and NF-κB is inhibited. (**B**) Following their NF-κB dependent expression MyD88, IRAK-1 and IRAK-2 undergo alternate splicing in certain tissues giving rise to myD88s, IRAK-1c and IRAK-2c/d which act in a dominant fashion to disrupt the signalosome complex and NF-κB activation. See text for details.

A common mechanism of negative feedback regulation of IL-1/TLR signalling pathways is the generation of dominant-negative variants of upstream receptor-proximal SC components via stimulus-induced expression and alternative splicing; examples include dominant-negative forms of the IRAK family of proteins and MyD88. IRAK-M is restricted in humans to monocytes/macrophages and is induced by TLR ligands including LPS [57]. IRAK-M lacks kinase activity [58] and acts as a negative regulator of IL-1R/TLR family signalling by trapping IRAK-1 and IRAK-4 in the receptor complex with MyD88 and blocking IRAK-4-mediated phosphorylation of IRAK-1; this prevents the association between IRAK-1 and TRAF-6 and inhibits downstream IKK/NF-κB activation (Figure 9A). The significance of this negative feedback is shown in IRAK-M*^−^*^/*−*^ macrophages, which secrete elevated levels of cytokines in response to IL-1R/TLR ligands and in IRAK-M*^−^*^/*−*^ mice, which exhibit enhanced intestinal inflammation following challenge with *Salmonella typhimurium.* IRAK-1c, a catalytically inactive IRAK-1 splice variant, is expressed in response to LPS in human monocytes and DC [59]. It retains the ability to associate with MyD88, IRAK-1/2 and TRAF6, but cannot be phosphorylated by IRAK-4 or undergo autophosphorylation, and acts in a dominant-negative manner to block signal transduction and NF-κB activation (Figure 9B). The murine IRAK-2 gene undergoes alternative splicing to generate four isoforms, two of which, IRAK-2c and IRAK-2d, inhibit NF-κB signalling via an undefined mechanism. The IRAK-2c promoter contains putative NF-κB binding sites and IRAK-2c expression is induced by LPS, identifying this isoform as a likely negative feedback regulator [60] (Figure 9B). Finally, LPS stimulation promotes the expression of a short splice variant of MyD88, MyD88s, which lacks the domain responsible for interaction with IRAK-4 [61]. MyD88s interacts with TLRs, IL-1R and IRAK-1, but cannot recruit IRAK-4 to the SC and so interferes with IRAK-4-mediated IRAK-1 phosphorylation and NF-κB activation [62] (Figure 9B).

### Deubiquitylating enzymes (DUBs)

**Figure 10.**
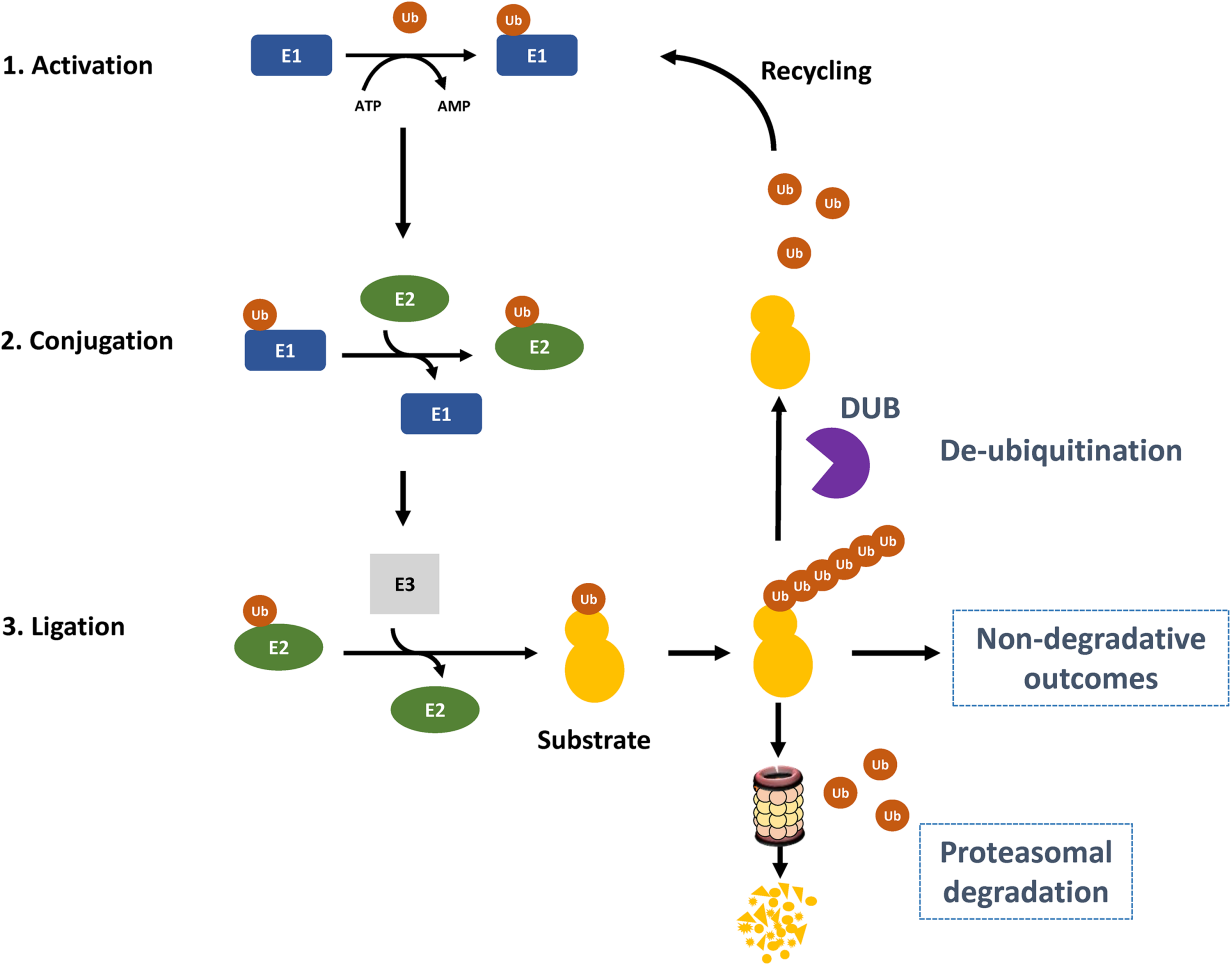
The activation, ligation and recycling of ubiquitin. A three-step enzymatic process leads to the ubiquitylation of a substrate protein. In the first stage, ubiquitin is activated in an ATP-dependent reaction by E1 ubiquitin-activating enzymes by way of the adenylation of the C-terminal carboxyl group. Next, ubiquitin is transferred via a thioester linkage to the active cysteine site on the E1, which releases AMP and pyrophosphate. In the second stage, ubiquitin is transferred to a sulfhydryl group on E2 ubiquitin-conjugating enzymes by a trans-thioesterification reaction. Finally, an E3 ubiquitin-ligase catalyses the transfer of ubiquitin to the substrate protein. This ubiquitylation process can be reversed by deubiquitylating enzymes (DUBs) which hydrolyse the isopeptide bond between ubiquitin and the substrate protein, or the peptide bond between ubiquitin molecules to remove the modification either partially or completely. Free ubiquitin produced by DUBs or by the UPS is then recycled to maintain a constant pool. See text for details.

Ubiquitylation, the reversible covalent attachment of ubiquitin (Ub) to proteins, plays fundamental roles in the regulation of the NF-κB pathway. The process of ubiquitylation proceeds through the stepwise, coordinated activity of Ub-activating enzymes (E1s), Ub-conjugating enzymes (E2s) and Ub ligases (E3s), and results in the covalent conjugation of the 76 amino acid ubiquitin protein via its C-terminal glycine carboxylic acid group to the epsilon amine of a lysine residue on the target protein (Figure 10). In turn, polyubiquitin chains of various linkage may be assembled through the conjugation of the C-terminus of a distal ubiquitin moiety to one of the seven lysine residues (K6, K11, K27, K29, K33, K48 and K63) or the N-terminal methionine (M1) within the proximal ubiquitin residue. Further complexity is introduced by the assembly of hybrid chains of mixed linkage, such as M1/K63 [63]. The fate of the ubiquitylated substrate is influenced by the nature of the polyubiquitin linkage. For example, K48 and K11 polyubiquitin chains typically direct proteins for proteasomal degradation, whilst K63 polyubiquitin chains act as docking sites for other proteins with specific ubiquitin-binding domains (UBD) and serve many functions including assembling signalling complexes and targeting proteins for autophagic degradation.

**Figure 11.**
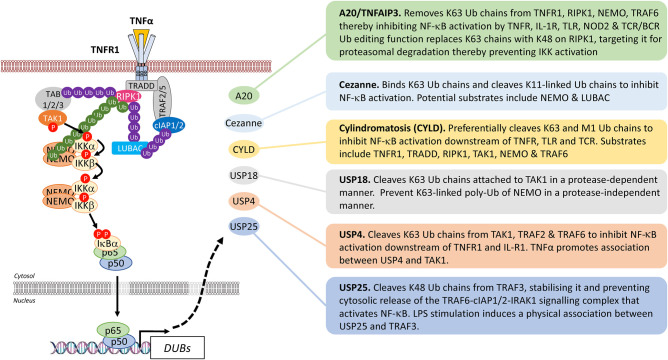
Deubiquitylases that function in feedback regulation of NF-κB activation. DUBs play important roles in feedback regulation of NF-κB activation. They are transcribed following NF-κB activation and catalyse deubiquitylation of select components of the upstream signalling complexes. In this way they disrupt activation of the IKK complex and so prevent further activation of NF-κB. They therefore serve a critical regulatory role, ensuring that NF-κB does not persist inappropriately. The DUB activities are summarised here and described in more detail in the text.

The essential roles ubiquitylation plays in the activation of NF-κB pathways has been discussed in detail elsewhere [64,65]. The focus here will be the reversal of ubiquitylation by deubiquitylating enzymes (DUBs). The human genome encodes ∼100 DUBs belonging to distinct families including: the ubiquitin specific proteases (USPs), the ovarian tumour (OTU) family, the Josephin domain family, the motif interacting with ubiquitin-containing novel DUB family (MINDY), the ubiquitin C-terminal hydrolases (UCHs) and the JAB1/MPN/Mov34 metalloenzyme domain family (JAMMs) [66,67]. DUBs are important to maintain the level of free Ub and can rescue proteins from Ub-mediated proteasomal degradation but they also exert fine, dynamic control on the assembly and activation of signalling complexes. Given the importance of ubiquitylation in propagating NF-κB activation, it is unsurprising that the DUBs are a major class of negative feedback regulators of NF-κB signalling (Figure 11).

#### A20 or TNFα-induced protein 3

A20, encoded by the *TNFα-induced protein 3* (*TNFAIP3*) gene, is an OTU family DUB and one of the most extensively characterised negative feedback regulators of canonical NF-κB signalling. Most cell types exhibit low basal A20 expression [68] but it is strongly induced at the transcriptional level by NF-κB following stimulation with TNFα, IL-1 or LPS [69,70]. The N-terminal OTU domain of A20 exhibits DUB activity towards K48-, K11-, and to a lesser extent, K63-linked polyubiquitin chains *in vitro* [71,72]. However, in cells A20 exhibits a strong preference for substrates bearing K63-linked chains [73,74], suggesting that A20 substrate specificity may be determined by interacting partners *in vivo*. Early studies demonstrated that overexpression of A20 inhibited NF-κB activation in response to proinflammatory stimuli [75,76]. Consistent with this, targets of A20 DUB activity include RIPK1 [74], TRAF6 [73], NEMO [77], RIPK2 [78], TNFR1 [74] and mucosa-associated lymphoid tissue lymphoma translocation 1 (MALT1) [79]. As such, A20 negatively regulates canonical NF-κB activation originating from TNFR, IL-1R, TLR, NOD2 and TCR/BCR stimulation.

A20 contains seven C-terminal Cys2-Cys2 zinc finger (ZF) domains [80] with ZF4 exhibiting an E3 ubiquitin ligase function, suggesting that A20 possesses dual ubiquitin-editing functions [74]. However, the A20 ubiquiting editing complex (AUEC)-associated E3 ligases, RNF11 and ITCH, may target K48-linked polyubiquitylation *in vivo* [81,82]. Regardless, A20 is proposed to sequentially substitute K63-linked polyubiquitin chains for K48-linked polyubiquitin, resulting in RIP1 proteasomal degradation which should destabilise the IKK-activating SC leading to inhibition of NF-κB signalling. Consistent with this, A20-deficient mice (A20*^−^*^/*−*^) exhibit a severe inflammatory phenotype and perinatal death due to multiple organ tissue inflammation and cachexia [83]. A20*^−^*^/*−*^ mouse embryonic fibroblasts (MEFs) also exhibit persistent hyperactivation of canonical NF-κB signalling, whilst conditional knock-out (KO) in various tissues has reinforced the importance of A20 in maintaining inflammatory and immunological homeostasis [84–86] and established A20 as a critical negative feedback regulator of NF-κB signalling.

However, the importance of A20 catalytic activity to its function as a negative feedback regulator has been questioned by the generation of DUB-defective and E3 ligase-defective A20 knock-in mice, which harbour inactivating point mutations in the catalytic site of the OTU domain (A20^OTU^) or ZF4 domain (A20^ZF4^), respectively, [87–89]. A20^OTU^ and A20^ZF4^ knock-in strains both exhibit increased responses to TNFα; however, unlike A20 KO mice, they are viable and exhibit none of the same spontaneous inflammatory phenotypes. Furthermore, MEFs derived from these mice display only mild [87,89], or even no [88] defects in TNFα or LPS-induced NF-κB activation. Finally, A20 KO MEFs reconstituted with either A20^OTU^ or A20^ZF4^ proteins exhibit approximately WT inhibition of TNFα-induced NF-κB activation [90]. Clearly, neither the DUB or E3 ligase activities are singly responsible for all of the regulatory functions of A20. A recent study wherein mice carrying three distinct targeted mutations of the zinc finger 7 (ZF7) domain of A20, which binds to M1-linked Ub chains, all developed digit arthritis with symptoms that are common to psoriatic arthritis, whereas mice that expressed point mutations in the OTU or ZF4 motifs did not exhibit this phenotype [91]. The similar phenotypes of the three mutant ZF7 mice lines suggested that ubiquitin binding is the major physiological function performed by A20's ZF7 motif and that this shared function prevents arthritis *in vivo*. As with the OTU and ZF4-deficient mice, these mice were viable and lived for upwards of 6 months, unlike the mice lacking A20 altogether. This group went on to hypothesise that ZF4 may compensate for the lack of ZF7, and that they work synergistically to optimise A20 binding to ubiquitylated signalling complexes. Indeed, these double mutant mice died within 3 weeks of birth and exhibited a severe phenotype similar to A20 knockout mice. Knock-in mice carrying both point mutations (A20^OTU/ZF4^) have yet to be characterised, but may more closely phenocopy A20 deficiency.

These knock-in mouse studies implicate the involvement of non-catalytic roles in the immunoregulatory function of A20. Indeed, two of the ZF domains in the C-terminus of A20 have been shown to act as UBDs; A20 ZF4 preferentially binds monubiquitin and K63-linked polyubiquitin [92], while ZF7 binds both M1-linked and K63-linked polyubiquitin [93,94]. Both of these domains facilitate recruitment of A20 to the IKK-activating SC. Binding of A20 ZF7 to long, unanchored K63-linked polyubiquitin chains has also been proposed to facilitate binding between NEMO and A20 within the SC. When bound to this NEMO-polyubiquitin complex A20 has been proposed to block the phosphorylation of IKK by TAK1 in a mechanism independent of its OTU domain catalytic residue (Cys103). The A20 ZF7 domain has also been proposed to enable A20 to compete with other ubiquitin-binding proteins, such as NEMO, for binding to M1-linked chains within the IKK-activating SC to inhibit downstream NF-κB in a manner independent of its DUB activity [95]. The mechanism has yet to be defined, but possibly involves protection of M1-linked chains from hydrolysis by other DUBs such as CYLD (see below). A20 also inhibits the activity of various E3 ligases, including TRAF2, cIAP1/2 and TRAF6 through physical blockade of their interaction with the E2 enzymes UbcH5c and Ubc13 [96]. At later stages in the signalling response (4–6 h), A20 also promotes K48-linked polyubiquitylation and proteasomal degradation of UbcH5 and Ubc13. Finally, a small fraction of cellular A20 localises to the lysosomal compartment in a ZF-dependent manner where it may targeting TRAF2 for lysosomal degradation [97,98]. It should be considered that the observation of these disparate mechanisms of action of A20 may reflect the use of different model systems; certain mechanisms may be dominant in different cell types exhibiting variations in endogenous A20 expression and at different time points.

The intrinsic enzymatic activity of A20 is also regulated via post-translational modifications. An important modification in the context of negative feedback regulation is the phosphorylation of A20 at S381 by IKKβ, which has been observed in IMCD, THP-1 and MEF cells following stimulation with TNFα or LPS stimulation [99]. This phosphorylation enhances A20 K63-linked DUB activity towards RIP1 and promotes ZF4-mediated substrate K48-linked ubiquitylation thus contributing further to the negative feedback regulation of NF-κB signalling [89]. In addition, assembly of the AUEC is regulated by phosphorylation following stimulation with cytokines. Specifically, IKKα-dependent phosphorylation of TAX1BP1 at S593 and S624 is essential for efficient termination of NF-κB signalling [100].

A20 function is also strongly influenced by protein interaction partners that, together with A20, constitute the A20 ubiquitin-editing complex (AUEC). Core members of this complex include Tax1-binding protein 1 (TAX1BP1), and the E3 ubiquitin ligases, RING finger protein 11 (RNF11) and ITCH, but the exact composition of the complex may vary depending on the stimulus and/or cell type. For example, A20-binding inhibitor of NF-κB 1 (ABIN-1), ABIN-2, ABIN-3 and YMER all interact with A20 under certain conditions [101,102]. Importantly, loss of either of the core components of the complex (TAX1BP1, ITCH and RNF11) disrupts A20 function and feedback inhibition of NF-κB signalling.

TAX1BP1 contains a UBD and functions as an adaptor within the AUEC, recruiting A20 to its K63-linked polyubiquitylated RIPK1 and TRAF6 substrates following TNFα and IL-1/LPS stimulation, respectively. TAX1BP1-deficient mice are hyperresponsive to inflammatory challenge, while TAX1BP1 KO MEFs display enhanced and prolonged NF-κB signalling following TNFα, IL-1 and LPS challenge. ITCH is a Nedd4 family HECT domain E3 ligase that is inducibly recruited to A20 through interaction with TAXBP1 and recruits A20 to RIPK1 for ubiquitin editing and degradation following TNFα stimulation [81]. Whether both ITCH and A20 ubiquitylate RIPK1 or if ITCH E3 ligase activity somehow promotes the E3 ligase activity of the A20 ZF4 domain is currently unclear. Consistently, ITCH KO MEFs also display enhanced and persistent signal-induced NF-κB activation. Meanwhile, RNF11 is a RING-type E3 ligase that inducibly interacts with TAXBP1, ITCH and RIPK1 upon TNFα/IL-1 stimulation [82]. siRNA-mediated knockdown of RNF11 results in enhanced and persistent signal-induced NF-κB signalling, while RNF11 overexpression inhibits NF-κB activation. RNF11 is required for A20 to interact with and inhibit RIPK1 but the role of RNF11 in A20 ubiquitin editing is currently unclear. However, given the functional importance of its PPXY motif, which mediates binding to ITCH, it may function as an adaptor for ITCH and/or regulate its catalytic activity.

The ABINs are also potentially important components of the AUEC. ABIN-1, -2 and -3 share four regions of high sequence similarity called ABIN Homology Domains (AHD). AHD1, AHD2 and AHD4 are common to all ABINs, while AHD3 is found only in ABIN-1 and ABIN-3 [103,104]. AHD1 is required for A20 binding, whereas AHD2 is responsible for polyubiquitin binding. Notably, AHD2 is also referred to as the ‘UBD in ABIN proteins and NEMO’ (UBAN) domain, due to its sequence homology with the UBD in NEMO [105]. ABIN-1 also contains a C-terminal NEMO-binding domain (NBD), similar to IKKα/β [77]. Each of the ABIN family members are implicated in the negative regulation of NF-κB signalling; their overexpression inhibits TNF, IL-1 and LPS-mediated activation of NF-κB. The UBAN, AHD1 and NBD domains have all been shown to be important for this function [77,105,106]. ABIN-1 and ABIN-3, but not ABIN-2, are up-regulated in response to NF-κB-activating stimuli (including TNFα and LPS), implicating these ABINs in negative feedback regulation [107,108].

Early studies implicated an A20-dependent mechanism for the inhibitory function of ABIN-1, whereby ABIN-1 acts as an adaptor to recruit A20 to its ubiquitylated target substrates. For example, ABIN-1, A20 and NEMO can interact as a complex and siRNA-mediated knockdown of ABIN-1 disrupts A20-dependent K63-linked deubiquitylation of NEMO, while siRNA-mediated knockdown of A20 impairs ABIN-1's NF-κB inhibitory activity [77]. However, considerable evidence also suggests ABIN-1 functions independently of A20. For instance, ABIN-1 mutants that are unable to bind A20 still inhibit NF-κB activation when overexpressed [104], while mutations that disrupt polyubiquitin binding also block the ability of ABIN-1 to inhibit TNFα-induced NF-κB activation. Furthermore, such UBAN mutants exert dominant negative effects on the NF-κB inhibitory function of WT ABIN-1, but not A20 [104]. This A20-independent model suggests that ABIN-1 competes with other NF-κB signalling components, such as NEMO, for polyubiquitin binding, thus preventing the signalling events required for NF-κB activation.

However, the importance of ABIN-1 in the negative feedback regulation of NF-κB signalling has been questioned by the generation of ABIN-1 KO mice [109]. ABIN-1-deficient MEFs exhibit normal NF-κB signalling in response to TNFα stimulation, although compensation by other ABIN family members has not been be ruled out. ABIN-1 KO mice die during late embryogenesis due to fatal TNFα-induced liver cell death and hypoplasia, suggesting that ABIN-1 has an important role in counteracting the cell-death inducing effects of TNFα. Surprisingly, ABIN-1-knockin mice expressing the polyubiquitin-binding defective mutant ABIN^D485N^ exhibit a strikingly different phenotype [106]. These mice are born normally but develop a lupus-like autoimmune diseases caused by hyperactive TLR-induced NF-κB signalling. Thus the importance of ABIN-1 as a negative feedback regulator of NF-κB signalling appears to be dependent on the signalling context.

ABIN-3 also interacts with NEMO and inhibits LPS-induced NF-κB activation downstream of TRAF6, but upstream of IKKβ [103]. However, its mechanism of action has not been defined. Similar to ABIN-1, deletion of the UBAN domain completely abolishes ABIN-3 NF-κB inhibitory activity, while deletion of AHD1 has only a minor effect suggesting its mode of action is independent of A20-binding [110].

#### Cellular zinc finger anti-NF-κB (Cezanne)

Cezanne is an OTU superfamily DUB with putative roles as a negative feedback regulator of NF-κB signalling. Cezanne is induced following TNFα stimulation and is recruited to activated TNFR SCs where it inhibits the accumulation of K63-linked polyubiquitylated RIPK1 complexes [111], although the exact NF-κB signalling component specificity of Cezanne has not been thoroughly characterised. DUB activity is important for this function as a catalytically inactive mutant is defective for RIPK1 deubiquitylation and NF-κB inhibition. Cezanne was the first DUB to be identified with a preference for cleaving K11-linked polyubiquitin chains [112]. K11 chains are implicated in NF-κB activation; for example, both cIAP1 and UbcH5 promote K11-linked non-degradative polyubiquitylation of RIPK1 in response to TNFα [113], while NEMO and LUBAC bind K11-linked chains during their recruitment to the TNFR SC [114]. Recently, the N-terminal ubiquitin-associated (UBA) domain of Cezanne was proposed to be essential for its ability to bind K63-linked chains, localise to activated TNFR SCs and inhibit canonical NF-κB activation [115].

However, the involvement of Cezanne in the regulation of canonical NF-κB activation has been questioned by the phenotype of Cezanne KO mice; MEFs and bone-marrow derived macrophages (BMDMs) derived from Cezanne KO mice exhibit no defects in TNFα/IL-1β-induced and LPS-induced NF-κB activation, respectively [116]. Instead, Cezanne KO mice exhibit hyperactivation of non-canonical NF-κB activation in response to agonistic anti-LTβR and anti-CD40 antibodies or BAFF. In response to non-canonical NF-κB stimulation Cezanne may deubiquitylate TRAF3 to prevent its proteasomal degradation. Certianly, non-canonical NF-κB signals induce Cezanne expression, highlighting a potential negative feedback role. Clearly, further work is necessary to resolve the function of Cezanne in NF-κB signalling pathways.

#### Ubiquitin carboxyl-terminal hydrolase or Cylindromatosis (CYLD)

CYLD is a USP superfamily DUB involved in the negative regulation of NF-κB signalling following stimulation of TNFR1, TCR and TLR receptors. CYLD exhibits a preference for K63 and M1 polyubiquitin chains, but also exhibits some *in vitro* activity towards K48 and K11 chains [117,118]. A wide range of NF-κB signalling components have been proposed as CYLD substrates, including NEMO [119], RIPK1 [120,121], TAK1 [122,123], TNFR1 [95], TRAF2 [119,124], TRADD [95], TRAF6 [125,126] and TRAF7 [126]. Like A20, CYLD may require ubiquitin-binding adaptor proteins to interact with its various substrates. For example, optineurin (OPTN) is important for CYLD binding to RIPK1 and its inhibitory effects on NF-κB signalling in response to TNFα [127].

Various CYLD-deficient/mutant mice confirm the important role of CYLD as a negative regulator of NF-κB signalling and inflammation. For example, CYLD KO mice exhibit increased inflammation and tumour formation in a colitis-associated cancer model, while lymphocytes and macrophages from these mice exhibit enhanced and prolonged stimulus-induced NF-κB signalling [119]. However, there are contradictory reports within the literature as to the function of CYLD as an endogenous inhibitor of basal NF-κB activation or as a signal-induced negative feedback inhibitor. For example, CYLD is constitutively expressed in most cell types, and its knockdown in HeLa cells results in constitutive ubiquitylation of TRAF2 and NF-κB activation. B cells and T cells from CYLD KO mice also exhibit constitutive NF-κB activation [122,128]. The endogenous DUB activity of CYLD has been proposed to be inhibited by rapid and transient IKK-dependent phosphorylation on multiple serine residues between amino acids 418-444 in response to TNFα, LPS and other proinflammatory cytokines [129]. These phosphorylation events prevent CYLD-mediated TRAF2 deubiquitylation, facilitating signal-induced NF-κB activation. However, the majority of other mouse studies have shown that CYLD-deficiency does not lead to spontaneous NF-κB activation, but rather to defects in the termination of signal-induced NF-κB activation [119,130]. Indeed, contrary to other reports, Zhang et al. observed no constitutive B cell NF-κB activation in independently derived CYLD-deficient mice [119]. Furthermore, NF-κB-dependent transcriptional up-regulation of CYLD in response to TNFα, IL-1β and bacterial pathogens has been reported in certain cell types [131], and CYLD protein interactions important for its activity are regulated in a signal-dependent manner [132]. Finally, in contradiction to the aforementioned study of CYLD phosphorylation, a report has suggested that CYLD S418 phosphorylation increases its DUB activity towards K63-linked polyubiquitin *in vitro* [133]. It is possible that CYLD constrains basal NF-κB activation and/or acts as a signal-induced feedback regulator of the IKK-activating SC under different biological contexts, perhaps dependent on the basal expression level of CYLD. Differences in the reported consequences of CYLD deficiency could also be due to varying compensation for the loss of CYLD function by other DUBs, such as A20.

Understanding the mechanism by which CYLD inhibits TNFR1-dependent NF-κB signalling has recently been advanced by the identification of the CYLD-interaction partner, SPATA2. Several reports have demonstrated that the interaction between CYLD and the E3 ligase subunit of the LUBAC complex, HOIP, is mediated by SPATA2 [132,134–136]. This CYLD/SPATA2/LUBAC complex assembles in the cytosol in the absence of stimulation and is recruited to the TNFR1 complex via interaction between LUBAC and K63-linked polyubiquitin chains within the SC following TNF stimulation. Here SPATA2 promotes CYLD DUB activity towards multiple SC components, including RIPK1, TRADD and TNFR1. However, there are varying reports regarding the effect of SPATA2-deficiency on TNF-induced NF-κB activation; some studies observed increased TNFα-induced NF-κB activation in cells lacking SPATA2 [132,136], while others observed no effect [134,135]. These differences are reminiscent of the heterogeneity observed in CYLD-deficient mice.

An independent feedback mechanism involving CYLD-mediated deubiquitylation of TAK1 has been proposed to restrict the inflammatory response to TNFα [123]. TNFα stimulation of bonemarrow derived macrophages stimulates the association of CYLD with ITCH, leading to the sequential K63-linked deubiquitylation, K48-linked ubiquitylation, and hence proteasomal degradation of TAK1 to terminate of the inflammatory response.

#### Other DUBs implicated in negative regulation of NF-κB signalling

A subset of USP superfamily members have also been implicated in the negative regulation of NF-κB signalling. For example, USP18 has been identified as a feedback inhibitor of TLR-mediated NF-κB activation in human macrophages/monocytes [137]. USP18 is induced following LPS stimulation and cleaves K63-linked polyubiquitin chains attached to TAK1 in a protease-dependent manner and inhibits K63-linked polyubiquitylation of NEMO in a protease-independent manner. USP4 has been proposed to remove K63-linked polyubiquitin chains from TAK1 [138], TRAF2 and TRAF6 [139] to inhibit NF-κB activation downstream of TNFR1 and IL-R1. There is currently no evidence that the expression of USP4 is induced by activators of NF-κB, but TNFα doesn promote association between USP4 and TAK1 [138]. Finally, USP25 has been proposed to restrict TLR4-dependent NF-κB signalling [140]. LPS stimulation induces a physical association between USP25 and TRAF3 and promotes the USP25-dependent cleavage of K48-linked polyubiquitin chains from TRAF3, which blocks its proteasomal degradation and the subsequent cytosolic release of the TRAF6-cIAP1/2-IRAK1 signalling complex that activates NF-κB. However, to date there is limited or no data from knockout mice to inform on the physiological roles of USP18, USP4 or USP25 in regulating NF-κB signalling.

### Interference with assembly/composition of the signalosome complex

A large number of other proteins have been implicated in the feedback inhibition of NF-κB signalling at the level of the IKK-activating SC. A common feature of these regulators is that they interfere with the composition and/or assembly of the SC, either through disrupting protein interactions or targeting signalling components for post-translational modification and/or proteasomal degradation. However, evidence for the involvement of many of these proteins in the negative regulation of NF-κB activation is heavily reliant on overexpression studies, which should be interpreted with caution given the known importance of protein stoichiometry to the normal function of NF-κB signalling. Overexpression artefacts are particularly common in the case of ubiquitin-binding proteins. This phenomenon is illustrated in the case of Optineurin, where *in vitro* overexpression/knock-down studies suggest a role as a negative feedback regulator of NF-κB which is not supported from *in vivo* functional studies.

Optineurin (OPTN) is a ubiquitously expressed ubiquitin-binding protein with a high degree of homology to NEMO. In cell based studies overexpression of OPTN inhibits TNFα-induced NF-κB activation, while siRNA-mediated knockdown increases basal and TNFα-induced NF-κB activity [141]. Two mechanisms have been proposed to explain this apparent inhibitory role of OPTN on NF-κB signalling; competition with NEMO for binding to ubiquitylated RIPK1 [142] or recruitment of CYLD to the SC [127]. Furthermore, OPTN expression is induced by TNFα in an NF-κB-dependent manner [141]. Collectively, these studies implicated OPTN as a negative feedback regulator of TNFα-induced NF-κB signalling. However, *in vivo* studies have failed to support this role. For example, OPTN KO mice develop normally and display no spontaneous inflammatory phenotypes, while OPTN KO MEFs exhibit no deregulation of TNFα or LPS-induced NF-κB activation [143]. Furthermore, normal TNFα/LPS/TCR/BCR-induced NF-κB responses are observed in knock-in mice expressing a point mutation in OPTN that completely abrogates polyubiquitin binding [144], and in mice lacking either the N-terminal TBK1-binding domain [145] or the C-terminal ubiquitin binding region [146]. These studies suggest the findings of the *in vitro* studies may reflect indirect effects of OPTN expression on NF-κB signalling through interference with other signalling pathways such as autophagy, where there is a well-defined role for OPTN [147].

The tripartite-motif (TRIM) family protein TRIM30α is a negative feedback regulator of TLR-induced NF-κB activation [148]. Its expression is induced specifically in lymphatic tissues by various TLR agonists, including LPS and CpG dinucleotide, in an NF-κB-dependent manner. TLR stimulation also promotes an interaction between TRIM30α and the TAK1-TAB2/3 complex. Subsequently, TRIM30α targets TAB2/3 for lysosomal degradation, which inhibits TAK1 activity and prevents TRAF6 autoubiquitylation; events important for downstream IKK/NF-κB activation. Consistent with these findings, siRNA-mediated TRIM30α knockdown in mice impairs LPS tolerance, while transgenic overexpression of TRIM30α confers resistance to endotoxic shock. TRIM38, another TRIM family protein, functions as a negative feedback regulator of TNFα, IL-1 and TLR-induced NF-κB activation. Its expression is induced in certain cell types, including mouse macrophages and RAW264.7 cells by TLR agonists in an NF-κB-dependent manner [149]. TRIM38 expression is also strongly indirectly induced by NF-κB signalling in the late phase of the inflammatory response to pathogenic agents by type I interferons [150]. TRIM38 has been reported to inhibit TLR3/4-induced NF-κB activation through K48-linked polyubiquitylation and proteasomal degradation of TRAF6 [149]. A separate study suggested that TRIM38 instead mediates its inhibitory effect on TLR3/4-induced NF-κB activation by targeting the adaptor protein TRIF for proteasomal degradation [151]. Late phase IFN-dependent induction of TRIM38 was proposed to inhibit TNFα/IL-1-induced NF-κB signalling through targeting of TAB2/3 for lysosomal degradation [151].

The Suppressor of cytokine signalling (SOCS) proteins are central regulators of microbial pathogen-induced inflammatory signalling. The SOCS family, consisting of eight members, SOCS 1-7 and CIS (cytokine-inducible SH2-containing protein), were originally identified as feedback inhibitors of cytokine-induced JAK/STAT signalling [152,153]. However, SOCS-1 and SOCS-3 have also been shown to act as negative feedback inhibitors of IL-1/TLR-induced NF-κB pathways. SOCS-1 and SOCS-3 are expressed in response to various inflammatory cytokines [153] but appear not to be direct targets of NF-κB. For example, the NF-κB target genes, IL-6 and IFN-γ can both promote the expression of SOCS-1 and SOCS-3 [153] at late stages in the NF-κB signalling response. One mechanism by which SOCS-3 acts as a negative regulator of IL-1-induced NF-κB signalling is through binding to TAK1 and TRAF6 to block their interaction, inhibit TRAF6 autoubiquitylation and inhibit TAK1 kinase activity. [154].

SOCS-1 and SOCS-3 are induced in dendritic cells (DCs) as tertiary response genes following TLR signalling [155]. TLR-induced NF-κB activation promotes IFN-family gene expression, which in turn promotes autocrine STAT1 signalling. Subsequently, STAT1 signalling is vital for the up-regulation of TAM (Tyro-3, Axl and MER) family receptor tyrosine kinases. Collectively, STAT1 and TAM-dependent signalling drive the expression of SOCS-1/3. In this context SOCS-3 acts on TRAF-6 and TAK1 to inhibit TLR-dependent NF-κB activation, while SOCS-1 acts on the TLR4 adaptor protein MyD88-adaptor-like (MAL) [156]. MAL is a critical adaptor that recruits cytosolic MyD88 to activated TLR4 receptors during the assembly of the so-called ‘Myddosome’ SC [157,158] and plays an important role in the phosphorylation of p65 within its transactivation domain (TAD) [159]. SOCS family proteins contain a SOCS box domain that enables them to recruit E3 ligase complexes to target substrates destined for proteasomal degradation [160]. Indeed, newly synthesised SOCS-1 binds to activated MAL, promoting its K48-linked polyubiquitylation and proteasomal degradation, thereby leading to inhibition of TLR-induced NF-κB signalling.

Sterile alpha- and armadillo-motif-containing protein (SARM) is the most recently identified of the five mammalian TIR-domain containing TLR adaptor proteins; the others being MyD88, MAL, TRIF and TRIF-related adaptor molecule (TRAM). The latter four adaptors have activating functions in TLR signalling, whereas SARM is a negative feedback regulator of TRIF-dependent NF-κB signalling [161]. SARM expression is rapidly induced within 1 h of LPS stimulation, although NF-κB-dependency has not been confirmed [162]. Newly synthesised SARM interacts with TRIF to inhibit downstream signalling [161] but the exact mechanism involved is unknown.

TNFR-associated factor 1 (TRAF1) is a member of the TRAF family of cytoplasmic adaptors, which transduce intracellular signals from receptors of the TNF-R superfamily. TRAF1 is unique amongst the seven TRAF family members due to its lack of an N-terminal RING finger domain that confers E3 ligase activity to the other members. TRAF1 is a well-defined NF-κB target gene whose expression is induced by proinflammatory cytokines, including TNFα, IL-1 and LPS [163]. However, unlike most other TRAF family members the exact role of TRAF1 in NF-κB signalling is unclear with both positive and negative roles in NF-κB signalling proposed. Supporting a negative role, TRAF1 overexpression inhibits canonical NF-κB activation in response to various stimuli [164], while the initial report of TRAF1 KO mice showed that TRAF1-deficient T cells are hyperresponsive to TNFα [165]. TRAF1 has also been shown to inhibit TRIF-dependent NF-κB signalling [166]. However, the majority of studies of lymphocyte signalling suggest a positive role for TRAF1 in NF-κB signalling [167–170]. In addition, the relative contribution of TRAF1 to the regulation of canonical versus non-canonical NF-κB signalling is unclear. For example, TRAF1 has been proposed to restrict non-canonical signalling in anti-CD3 activated T cells [171], and to bind and stabilise NIK to promote non-canonical NF-κB activation in a positive feedforward mechanism downstream of TNFα-induced canonical signalling [172]. Recently, a novel negative feedback role for TRAF1 in TLR-induced NF-κB signalling has been identified [173]. TRAF1 binds to all three subunits of the LUBAC complex (SHARPIN, HOIL and HOIP) to block the SC recruitment and linear ubiquitylation of NEMO. Consistent with these findings, TRAF1-deficient mice exhibit increased susceptibility to LPS-induced septic shock, while TRAF1 KO BMDMs derived from these mice display significantly enhanced LPS-induced NF-κB activation.

DNA damage represents an atypical signal that can trigger NF-κB activation that has been comprehensively reviewed [174]. In brief, genotoxic stress triggers the SUMOylation and nuclear accumulation of an ‘IKK-free’ form of NEMO [175]. In turn, NEMO associates with and is phosphorylated at S85 by the DNA damage-activated nuclear kinase ATM (ataxia telangiectasia mutated), leading to NEMO's monoubiquitylation and nuclear export in a complex with ATM [176]. This ATM-NEMO complex subsequently activates IKK in the cytoplasm to induce NF-κB activation [177,178]. The E3 ligase mediating the SUMOylation of NEMO in response to DNA damage is protein inhibitor of STAT y (PIASγ) [179]. In the same way that DUBs catalyse the reversal of ubiquitylation, members of the Sentrin/SUMO-specific protease (SENP) family catalyse the removal of SUMO conjugates from target proteins [180,181]. A negative feedback mechanism involving the SENP family member SENP2 attenuates the pro-survival NF-κB signalling response to genotoxic stress [182]. SENP2 expression is selectively up-regulated by NF-κB activated in the context of genotoxic stress due to the involvement of NF-κB promoter recruitment and ATM-dependent histone methylation of SENP2 promoter NF-κB sites. In turn, SENP2 deSUMOylates NEMO to inhibit further NF-κB activation.

## Regulation of IKK kinase activity as a feedback mechanism

Comparatively few negative feedback mechanisms have been identified that directly target the IKK kinase subunits, IKKα and IKKβ. This is perhaps surprising given their central role as the master regulators of NF-κB signalling; all NF-κB pathways identified to date require the activity of at least one of the IKKs. Phosphorylation of the activation loop serine residues, S176/ S180 and S177/S181 in IKKα and IKKβ, respectively, is essential for the activation of IKK kinase activity. These phosphorylation events are rapid and transient, occurring within 5 min of receptor stimulation and peaking after 30–60 min before returning to resting levels. In other kinase signalling pathways the effector kinase often phosphorylates upstream components to inhibit signal propagation; for example, in the RAF-MEK-ERK kinase cascade, ERK can phosphorylate RAF and MEK to inhibit pathway activation [183]. There have been few reports of such direct phosphorylation of upstream components by the IKKs as a feedback mechanism. A notable exception is in the non-canonical pathway where stabilisation of NIK leads to phosphorylation and activation of IKKα. As well as phosphorylating p100, IKKα phosphorylates S809, S812 and S815 within the C-terminus of NIK to promote its proteasomal degradation [91] (Figure 6B); in the absence of IKKα, NIK expression accumulates continuously with time far beyond that observed in wild type cells. In this way, IKKα activation ensures down-regulation of its own activating kinase and this is a common homeostatic feedback mechanism.

Several phosphatases have been proposed to dephosphorylate and inhibit IKK kinase activity, including protein Phosphatase 2Cβ (PP2Cβ) [184], protein phosphatase 2A (PP2A) [185], protein serine/threonine phosphatase 2Ceta-2 (PP2Ceta-2) [186]; protein phosphatase 1 (PP1) [187,188], protein phosphatase 4c (PP4c) [189] and protein phosphatase 1A, magnesium-dependent, alpha isoform/ protein phosphatase 1A, magnesium-dependent, beta isoform (PPM1A/PPM1B) [190]. It is likely that there are cell type/stimulus-specific differences in these IKK-directed phosphatase activities. Furthermore, some phosphatases are proposed to limit basal IKK phosphorylation/activation, while others may participate in negative feedback loops to restore signal-induced IKK phosphorylation to basal levels. Examples of the latter include PP4c and PPM1A/PPM1B. The PP4c Ser–Thr phosphatase regulatory subunit PP4R1 was identified in a siRNA screen for negative regulators of TCR-dependent NF-κB activation in T lymphocytes [189]. TCR stimulation drives the expression of PP4R1 and promotes its association with the IKK complex, where it recruits the phosphatase subunit, PP4c, to interact with IKKα/β and catalyse their dephosphorylation. Accordingly, PP4R1 knockdown causes enhanced and prolonged TCR-induced IKK activation and T cell hyperactivation. Meanwhile, a functional genomic screen identified the Ser/Thr phosphatases, PPM1A and PPM1B as potential IKKβ phosphatases [190]. PPM1A and PPM1B were found to transiently associate with and dephosphorylate IKKβ following its TNFα-induced phosphorylation.

A few other regulatory mechanisms directly targeting the IKK kinases themselves have been identified. For example, the NOD-like receptor (NLR) family member, NLR family CARD domain containing 5 (NLRC5) is a negative feedback regulator of TNFα/IL-1/LPS-induced NF-κB activation that interacts with IKKα and IKKβ to block their phosphorylation [191]. NLRC5 expression is indirectly induced by LPS-dependent NF-κB activation via the NF-κB target gene, IFN-γ [192]. NLRC5 binds to the kinase domains of IKKα/β and physically blocks the binding of NEMO to IKK due to its large size [191]. Consistent with these findings, NLRC5 deficient MEFs/BMDMs exhibit enhanced LPS-induced IKK phosphorylation, NF-κB activation and proinflammatory cytokine gene expression. Another NOD-like receptor family member, NLR family member X1 (NLRX1), has been linked to the negative regulation of TLR-mediated NF-κB activation through inhibitory binding to the IKK complex [193]. Under basal conditions NLRX1 associates with TRAF6. Following LPS stimulation, NLRX1 undergoes rapid K63-linked polyubiquitylation, dissociates from TRAF6 and interacts with the kinase domain of phosphorylated/active, but not unphosphorylated/inactive IKKβ, to promote its dephosphorylation. Consequently, siRNA-mediated knockdown of NLRX1 enhances LPS-induced IKK phosphorylation and NF-κB dependent gene expression and increases susceptibility of septic shock in mice.

A variety of other proteins have been shown to dynamically associate with the IKK complex following TNFα stimulation to inhibit IKK activity and downstream NF-κB activation. For example, human coilin-interacting nuclear ATPase protein (hCINAP) associates with IKKα/β in response to TNFα stimulation and serves as an adaptor to recruit the phosphatase, PP1, to dephosphorylate IKK [188]. In addition, Ring finger protein 8 (RNF8) associates with IKKs following TNFα stimulation and inhibits IKKα/β phosphorylation and subsequent NF-κB activation [194].

A unique feedback strategy operating at the level of IκB phosphorylation, without directly influencing IKK activity, is exemplified by the long non-coding RNA (lncRNA), NF-κB interacting long non-coding RNA (NKILA) [195]. LncRNAs are a class of non-protein-coding transcripts of greater than 200 base pairs in length that regulate gene expression at multiple levels, typically through acting as signals, decoys, guides and scaffolds [196]. NKILA is up-regulated in breast cancer cells in response to LPS, IL-1β and TNFα in an NF-κB-dependent manner [195]. In turn, NKILA stably interacts with the NF-κB:IκBα complex in the cytoplasm and directly inhibits basal and stimulus-induced IKK-dependent phosphorylation of IκBα by masking the S32/S36 phosphorylation sites in IκBα. Decreased NKILA expression is correlated with high basal NF-κB activity in invasive breast cancer and is associated with poor patient prognosis.

## Inhibition of NF-κB-dependent transcriptional responses

A host of feedback mechanisms that impinge on the NF-κB subunits themselves have been identified. These mechanisms include: [1] interference with NF-κB DNA-binding and/or nuclear localisation; [2] regulation of interaction with other transcriptional cofactors; [3] inhibition of transactivation through phosphorylation and turnover and; [4] clearance of NF-κB subunits from promoter DNA. Regardless of the stimulus, all canonical NF-κB pathways are activated following IKK-dependent phosphorylation and proteasomal degradation of IκB proteins, which liberates NF-κB subunits to accumulate in the nucleus and bind to κB motifs in target gene promoters. Subsequent NF-κB-dependent reexpression of IκB proteins, such as the prototypical member IκBα, represent the best-understood examples of negative feedback loops involved in the global down-modulation of canonical NF-κB signalling pathways. A range of other mechanisms act within the nucleus on transcriptionally active NF-κB subunits already bound to individual target gene promoters and thus typically terminate the NF-κB response in a gene-specific manner. These feedback mechanisms play an important role in fine tuning the kinetics of the inflammatory gene regulatory program.

### Interference with NF-κB DNA-binding and/or nuclear localisation by the IκB inhibitor proteins

There are currently nine recognised IκB inhibitor proteins split into three groups: the ‘classical’ IκB proteins, IκBα, IκBβ and IκBε; the ‘precursor’ IκB proteins, p100 and p105; and the ‘atypical’ IκB proteins, Bcl-3, IκBζ, IκBNS and IκBη. IκBα is one of the earliest genes to be expressed following NF-κB activation, being completely resynthesized within 1 h following its stimulus-induced degradation [197]. Consistent with the importance of this IκBα-dependent feedback mechanism, IκBα-deficient mice exhibit neonatal lethality, chronic up-regulation of diverse NF-κB target genes and enhanced granulopoiesis and dermatitis [198,199]. Interestingly, the defects observed in IκBα-deficient mice can be rescued by placing the IκBβ coding sequence under control of the IκBα promoter, suggesting that the biochemical properties of the IκB protein are less important than the kinetics of IκB resynthesis [200].

**Figure 12.**
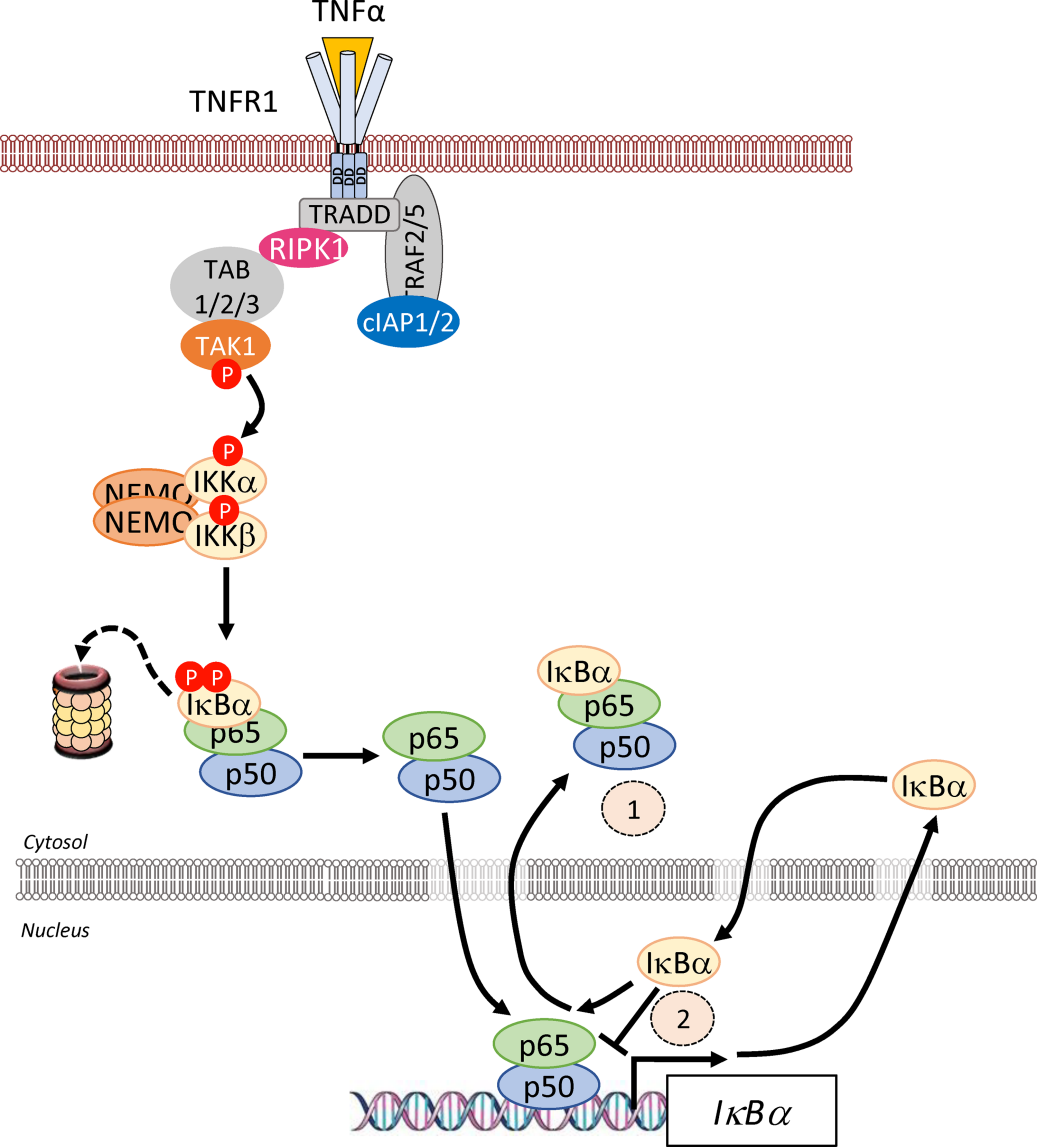
Inhibition of NF-κB-dependent transcriptional responses by IκB proteins. There are two main mechanisms by which IκBα is thought to terminate early phase NF-κB signalling. **1.** Newly synthesised IκBα may shift the equilibrium of transcriptionally active NF-κB dimers from the nucleus into the cytoplasm by dynamic shuttling thanks to the presence of nuclear export signal (NES) on IκBα and a nuclear localisation signal (NLS) on p50 that is not completely masked by IκBα. Once there, degradation of IκBα shifts the equilibrium back to the nucleus and so on. **2.** Newly synthesised IκBα may inhibit NF-κB activity by preventing it from interacting with κB sites on DNA. See text for details.

Newly synthesised IκBα is thought to terminate the early phase of NF-κB signalling via two main mechanisms. First, *de novo* IκBα shifts the equilibrium of canonical NF-κB subunit localisation from the nucleus to the cytoplasm. The classical conception of IκB proteins sequestering NF-κB proteins in the cytoplasm may not be entirely accurate; complexes of IκBα and p50:p65 have been shown to dynamically shuttle between the cytoplasm and the nucleus of resting cells due to the presence of a nuclear export signal (NES) in IκBα and a nuclear localisation sequence (NLS) in p50 that is incompletely masked by IκBα (the NLS of p65, however, is masked) [201–203]. Consequently, degradation of IκBα shifts the steady-state equilibrium of NF-κB distribution towards the nucleus, while newly synthesised IκBα shifts this equilibrium back towards the cytoplasm. Second, considerable *in vitro* evidence indicates that *de novo* expressed IκBα inhibits NF-κB activity by disrupting its interaction with DNA [204] (Figure 12). Early experiments showed that IκBα competes efficiently with DNA for binding NF-κB [205], leading to suggestions that IκBα removes NF-κB from the DNA via an ‘active dissociation’ mechanism. Biophysical approaches have confirmed that IκBα enhances the dissociation rate of NF-κB (p50/p65) from the κB sites in DNA in an efficient and concentration-dependent manner [206]. The structurally disordered ankyrin repeats (ARs) 5 and 6 of the IκBα ARD are required for IκBα to ‘strip’ NF-κB from DNA; this region folds upon binding to NF-κB enabling the negatively charged C-terminal PEST sequence of IκBα to displace bound DNA [207]. This enhanced dissociation rate is thought to increase the effectiveness of *de novo* IκBα in mediating transcriptional repression even prior to accumulation of IκBα to levels that are sufficiently high to compete effectively for κB sites in the DNA (i.e during the onset of resynthesis).

IκBε also plays an important role in feedback inhibition of NF-κB signalling. Like IκBα, IκBε expression is strongly induced by pro-inflammatory cytokines in an NF-κB-dependent manner [208,209] and IκBε also undergoes stimulus-induced IKK-dependent phosphorylation and β-TrCP-dependent proteasomal degradation, although the kinetics of IκBε degradation are slower than IκBα [210]. IκBε inhibits NF-κB activity via similar mechanisms as IκBα, although nuclear import of IκBε is less efficient than IκBα [211]. Interestingly, cells depleted of all three classical IκB proteins, IκBα, IκBβ and IκBε (termed 3KD cells) exhibit only small increases in nuclear p65 under basal conditions compared with WT cells [212]. This small increase drives constitutive activation of NF-κB target genes, such as IL-6 and iNOS, in the absence of stimulus, indicating that the classical IκB proteins are essential for preventing NF-κB DNA binding in the basal state. 3KD cells are severely defective in TNFα- and LPS-induced NF-κB activation compared with WT, indicating that classic IκB molecules are also essential for signal responsiveness.

The cytoplasmic sequestering function of the classical IκBs for NF-κB subunits can be compensated by the precursor IκB proteins, particularly p100. Unprocessed p100 cytoplasmically sequesters not only its processed NF-κB subunit product, p52, which is liberated following activation of the non-canonical NF-κB pathway, but also all other NF-κB subunits (p50, p65, c-Rel and RelB) through the formation of large tetrameric ‘kappaBsomes’ [213,214]. These complexes interact with NF-κB subunits through dimerisation between the Rel-homology domain (RHD) domains of individual subunits and interactions between p100 ankyrin repeats and preformed NF-κB dimers. In turn, non-canonical signals regulate the release of preformed canonical NF-κB dimers, such as p65:p50 from these kappaBsomes [37]. Importantly, proinflammatory cytokines induce the late-phase expression of the *NFKB2* gene encoding p100 in an NF-κB-dependent manner [215]. Collectively, these properties impart p100 with negative feedback functions in the attenuation of prolonged canonical NF-κB activities. Furthermore, the irreversibility of the p100 negative feedback loop (p100 does not undergo canonical stimulus-induced processing) combined with its slow induction renders it dominant over the faster acting IκBa and IκBε at late stages of sustained signalling. For example, stimulus-induced p100 exerts negative feedback on p65:p50 dimers to attenuate prolonged TLR-induced canonical NF-κB signalling [216] and also exerts late acting negative feedback to dampen TCR-induced NF-κB signalling and IL-2 production [217]. p100 has a proposed NES and some LPS/TNFα-induced p100 nuclear translocation has been observed, suggesting that p100 may also execute its negative feedback functions within the nucleus [218,219].

The atypical IκB family consists of BCL-3, IκBζ, IκBNS and IκBη and exhibit a number of properties that distinguish them from classical IκB proteins. For example, their basal expression is typically low and they are not degraded following pathway activation [220]. Rather, their expression is strongly induced in an NF-κB-dependent manner and, unlike classical IκB proteins, they can function within the nucleus as positive or negative feedback regulators of NF-κB-dependent gene expression in a context-specific manner. For example, BCL-3 contains a TAD, but its function as a positive or negative feedback regulator of NF-κB signalling is heavily dependent on its post-translational modification. BCL-3 is strongly induced by LPS in an NF-κB-dependent manner [221]. In its unphosphorylated form, BCL-3 functions as a feedback inhibitor of NF-κB-induced gene expression by binding to and inhibiting the ubiquitylation and degradation of chromatin-bound transcriptionally repressive p50 homodimers, thus preventing promoter replacement with transcriptionally active NF-κB dimers [222,223]. BCL-3 may also promote the recruitment of transcriptional repressors, such as HDAC-1, -3 and -6 [224] and may function as a classical IκB protein by displacing p50 and p52-containing dimers from active promoters. Consistently, BCL-3 deficient mice display hyper-responsiveness to LPS stimulation [222]. However, BCL-3 is phosphorylated in cells by AKT, ERK2 and IKKα/β, with the result in all cases being an increase in its transcriptional activation potential in p50/p52 dimers [225].

A negative feedback role in NF-κB signalling has also been proposed for IκBζ. In murine macrophages IκBζ is induced by LPS and IL-1β, but not TNFα [226,227] and IκBζ expression requires both NF-κB-dependent transcriptional activation and stimulus-specific stabilisation of its mRNA [228]. Human IκBζ is strongly induced in response to IL-1β and TNFα in HeLa and MCF-7 cancer cells [229]. Newly synthesised IκBζ localises to the nucleus where it binds to p65 and p50, inhibiting their DNA binding and transcriptional activity [229]; indeed, overexpression of IκBζ inhibits TNFα induction [230]. The mechanism has not been clearly defined, however IκBζ colocalises with HDAC5 and the corepressor SMRT in matrix-associated deacetylase nuclear bodies, pointing to a possible role for IκBζ in chromatin remodelling. In human cells, mRNAs of IκBζ and IκBα are induced with similar kinetics, suggesting that IκBζ represents an additional rapid negative feedback mechanism for the prompt termination of NF-κB transcriptional responses [229]. It is important to note that, like BCL-3, IκBζ also plays a positive role in the regulation of a subset of TLR-dependent genes, such as IL-6 and IL-12p40 [26].

The atypical IκB family member, IκBNS has also been proposed to function in a negative feedback loop to limit inflammatory responses in macrophages [231]. Induced by LPS stimulation, IκBNS inhibits the expression of a subset of late TLR-induced genes such as IL-6, IL-18, G-CSF and IL-12p40, possibly through association with promoter-bound p50 dimers and enhancement of their DNA-binding activity and/or promotion of p65 degradation [231,232]. Consistent with these findings, IκBNS-deficient mice are hypersensitive to LPS-induced septic shock and colitis-associated inflammation [231].

Notably, IκB-mediated negative feedback loops are important in regulating oscillations of NF-κB nuclear localisation and activity [233,234]. Oscillatory NF-κB dynamics, observed at the single-cell level, arise from the intrinsic time delays between activation of the signalling pathway and induction/accumulation of the proteins mediating each feedback loop [235]. The role of these oscillations is highlighted by the fact that changes in the pattern of pathway stimulation using pulses of TNFα alter NF-κB signalling dynamics and, in turn, drive unique transcriptional responses [236]. Importantly, the stimulus-dependent induction of IκBε is delayed by ∼45 min relative to IκBα [208,210,237]. As a result, the negative feedback loops mediated by IκBα and IκBε are in antiphase, such that IκBε-dependent feedback dampens IκBα-mediated oscillations in NF-κB activity during sustained pathway stimulation [237].

### Regulation of NF-κB interaction with other transcription factors

Numerous transcription factors act within the nucleus to inhibit the activity of NF-κB subunits. Many of these inhibit NF-κB transcriptional responses by regulating interactions of NF-κB subunits with co-activators or co-repressors. The bZip family transcription factor, activating transcription factor 3 (ATF3), is induced by LPS stimulation in an NF-κB-dependent manner in macrophages [238]. Following LPS stimulation, ATF3 interacts with p65 and recruits HDAC1 to p65 transcriptional complexes at the promoters of specific genes where it deacetylates and promotes dissociation of p65 from the DNA [238,239]. siRNA-mediated knockdown of ATF3 enhances the LPS-induced expression of various NF-κB target genes, such as *iNOS*, *IL-1β*, *IL-6*, *IL-12β* and *IκBζ*.

The transcription factors, Twist-1 and -2 are induced in MEFs by TNFα in an NF-κB dependent manner [240] and associate with p65 at the promoters of certain genes, including *TNFα* and *IL-1*β, inhibiting promoter activity by interfering with p65 transactivation. Whilst the exact mechanism remains to be defined, Twist-2 KO mice exhibit enhanced expression of proinflammatory cytokines and perinatal death from cachexia, while Twist-2 KO MEFs exhibit enhanced sensitivity to TNFα-induced apoptosis, consistent with the proposed repressive functions for these transcription factors.

The early response gene, immediate early response 3 (IER3), is induced by proinflammatory cytokines in an NF-κB-dependent manner [241] and mediates feedback inhibition of NF-κB activation via two different mechanisms. First, IER-3 attenuates the expression of 19S proteasomal components, Rpn10 and Rpn2, to interfere with the assembly and activity of the 26S proteasome, which in turn blocks the proteasomal degradation of polyubiquitylated IκBα [242,243]. Second, newly synthesised IER-3 directly interacts with the TAD of p65 bound to the promoter of certain NF-κB target genes, including *BCL2*, *cIAP1* and *cIAP2* to inhibit p65 transactivation and expression of these genes [244]. The mechanism of inhibition could involve steric competition of IER-3 with essential co-activators for binding to the p65 TAD, or interference with post-translational modification of p65, e.g. S536 phosphorylation [242].

The aryl hydrocarbon receptor (AhR) transcription factor mediates a negative feedback loop to inhibit LPS-induced NF-κB-dependent cytokine expression [245]. In macrophages, AhR expression is induced by LPS in an NF-κB-dependent manner and an AhR-STAT1 complex associates with p65:p50 dimers at the promoter regions of proinflammatory cytokines, inhibiting NF-κB transcriptional activity by an as yet undefined mechanism; Consistent with these findings, AhR-deficient mice exhibit enhanced sensitivity to LPS-induced septic shock.

### Inhibition of transactivation through phosphorylation and turnover

A common theme in the negative regulation of nuclear NF-κB activity is the modulation of NF-κB post-translational modifications. Site-specific NF-κB phosphorylation may promote or inhibit NF-κB activity through control of interactions with other factors, modulation of transcriptional activity and/or protein stability. However, for many of the NF-κB phosphorylation sites, there is conflicting evidence on biological outcome and this is exemplified by S536 in the TAD of p65. IKKα-dependent phosphorylation of S536 has been proposed as a major mechanism of pathway inhibition and resolution of inflammation in macrophages through the enhanced turnover of p65 and removal from proinflammatory gene promoters [246]. These findings are supported by the recent generation of an S534A- (mouse equivalent of human S536) knock-in mouse model, which exhibited modest increases in the late-phase expression of LPS-induced genes commensurate with increases in p65 stability [247]. However, various studies have also identified a positive role for S536 phosphorylation in NF-κB transcriptional responses. For example, S536 phosphorylation has been proposed to enhance transcriptional transactivation through increased CBP/p300 binding and acetylation of p65 at K310 [248]. Furthermore, the WIP1 phosphatase has been proposed to act as a negative regulator of NF-κB signalling by directly dephosphorylating p65 phosphorylated at S536 [249]. These apparently opposing effects of S536 phosphorylation need not be a conflict for two main reasons. First, since S536 phosphorylation can recruit both CBP/p300 and an E3 ligase complex they are hardly likely to bind to the same S536 phosphorylated p65 molecule at the same time; so distinct pools of S536 phosphorylated p65 likely exisit with distinct binding partners, perhaps determined by other PTMs. Second, the kinetics of recruitment of p300 or the E3 ligase complex that targets turnover of p-S536-p65 may provide a ‘window’ during which p300 binding can enhance p65 activity to drive gene expression before p65 turnover. The linking of an activating phosphorylation event to turnover of the protein concerned in this way is a common homeostatic motif in biology that ensures that signal pathway activation is kept under careful control.

**Figure 13.**
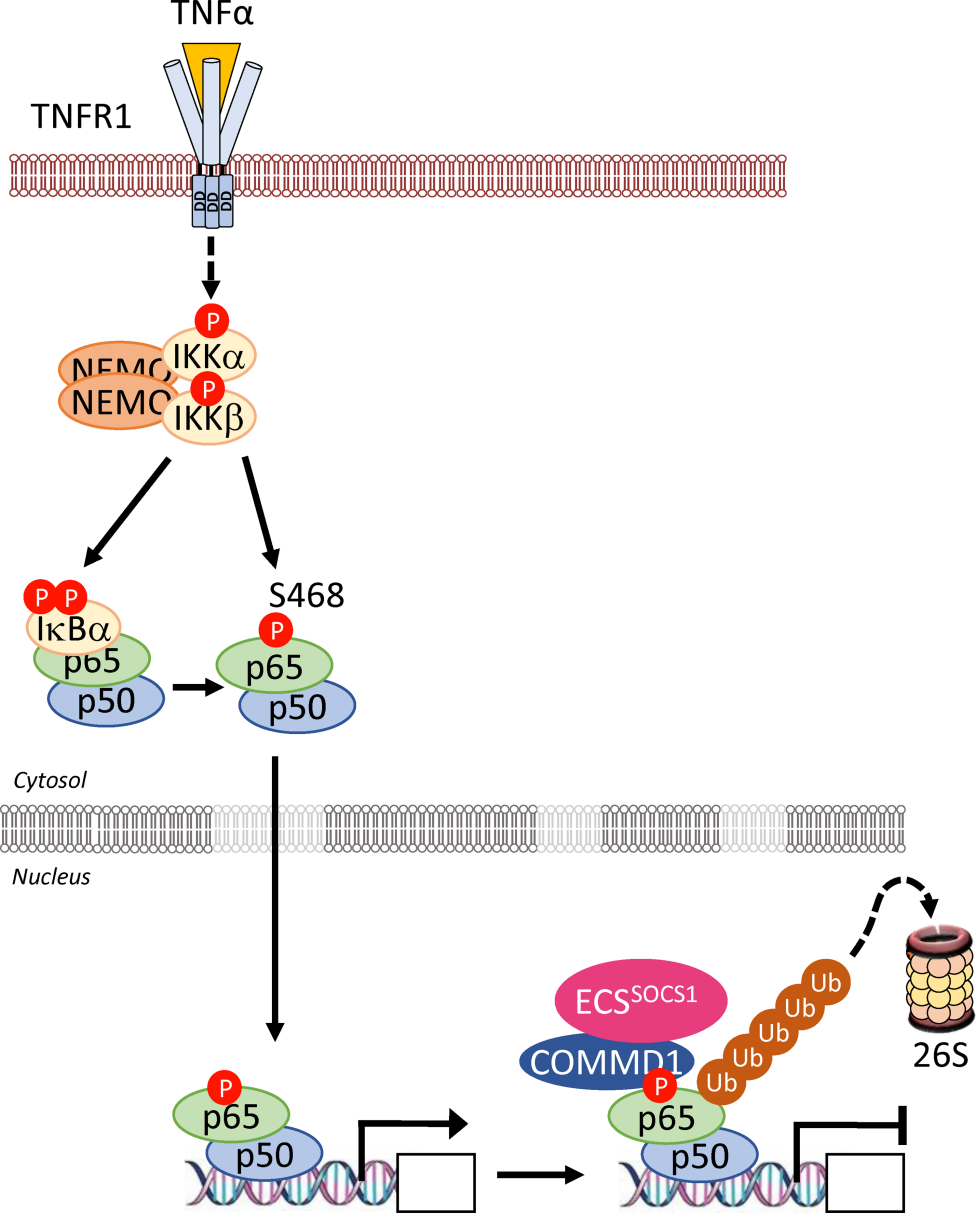
Inhibition of NF-κB activity via turnover of subunits. COMMD1 binds to p65 when it is phosphorylated at Ser 468 by the IKK complex. COMMD1 then recruits ECS^SOCS1^ E3 ubiquitin ligase complex to polyubiquitylate DNA-bound p65 subunits of NF-κB with K48 linkages. This leads to the proteasomal degradation of p65 and removes it from DNA, limiting the expression of NF-κB target genes. See text for details.

Another example involves the COMMD1-ECS^SOCS1^ complex. Early experiments with IκBα-deficient cells revealed the importance of chromatin-bound p65 polyubiquitylation and proteasomal degradation for termination of the late NF-κB transcriptional response [250]. Copper metabolism domain containing 1 (COMMD1) is involved in this process; COMMD1 deficiency results in the prolonged nuclear accumulation of p65 and enhanced and sustained expression of a subset of NF-κB-dependent genes following TNFα stimulation [251,252]. TNFα stimulation leads to the phosphorylation of p65 at S468, by IKKβ and/or IKKε [253,254], which promotes the recruitment of COMMD1 and the ECS^SOCS1^ E3 ligase component, Cullin 2 to chromatin-bound p65 [255]. The ECS^SOCS1^ complex consists of Elongins B and C, Cullin 2 and SOCS1 [256]. The interaction between COMMD1 and Cullin 2 is TNFα-inducible and COMMD1, in turn, stabilises the binding of p65 to SOCS1 [252]. COMMD1 thus acts as a scaffold to mediate interaction between the E3 ligase and its substrate. Collectively, this complex promotes the K48-linked polyubiquitylation of chromatin-bound p65, leading to its proteasomal degradation and clearance from a subset of target genes [255] (Figure 13). The histone acetyltransferase GCN5 has also been shown to be important in this process and interacts with COMMD1 and p65 in a manner dependent on prior phosphorylation of p65 at S468 [257].

Finally, an example of an indirect modulator of NF-κB phosphorylation is Tribbles homolog 3 (TRIB3). TRIB3 expression is induced in response to TNFα stimulation in an NF-κB-dependent manner and TRIB3 acts as a feedback inhibitor of NF-κB-dependent transcription by binding to p65 in the nucleus and blocking its stimulatory phosphorylation by protein kinase A [258].

### Recruitment of repressors and clearance of NF-κB subunits from promoter DNA

**Figure 14.**
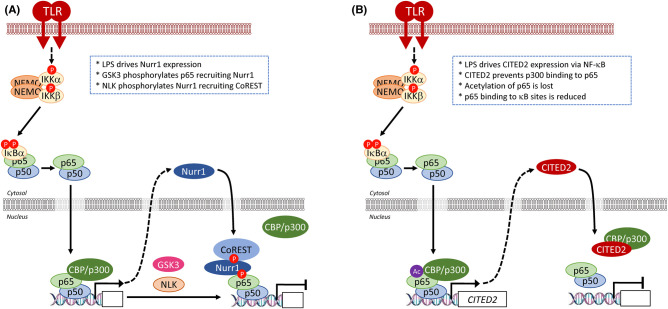
Recruitment of repressors leads to termination of transcriptional responses. (**A**) The orphan nuclear receptor Nurr1 is expressed by microglia following stimulation of the NF-κB pathway by LPS. Phosphorylation of p65 at S468 by GSK3β then acts as a docking site for Nurr1 interaction in the nucleus. Nurr1 then recruits the CoREST transcriptional co-repressor complex to DNA bound p65 subunits. CoREST is composed of chromatin-modifying enzymes which terminate NF-κB transcription. (**B**) In macrophages CITED2 expression is induced by LPS stimulation in an NF-κB-dependent manner. CITED2 localises to the nucleus where it interacts with the NF-κB coactivator p300, thereby impairing p65 acetylation, inhibiting retention of p65 at target promoters and reducing gene expression. See text for details.

Phosphorylation of NF-κB subunits may act as a trigger for the recruitment of regulators that terminate the transcriptional response is seen with Nurr1, an orphan nuclear receptor which regulates p65-dependent transcription to protect dopaminergic neurons from inflammation-induced cell death [259]. LPS stimulation induces Nurr1 expression in microglia and promotes the GSK3β-dependent phosphorylation of p65 at S468, which subsequently acts as a docking site for interaction with Nurr1 in the nucleus. In turn, Nurr1 recruits the CoREST transcriptional corepressor complex to promoter-bound p65 subunits. The CoREST complex consists of numerous chromatin-modifying enzymes, including a histone demethylase, histone methyltransferase G9a, lysine-specific demethylase (LSD1) HDAC 1 and 2, which cooperate to terminate the p65 transcriptional response and prevent chronic neurotoxic inflammation (Figure 14A).

Another common strategy utilised in the feedback termination of NF-κB transcriptional responses is the clearance of NF-κB subunits from promoter DNA. There are two non-mutually exclusive mechanisms by which this is achieved: promoter displacement and proteasomal degradation of NF-κB subunits [250]. In addition to the recruitment of COMMD1 and the ECS^SOCS1^ E3 ligase complex (see above), a second nuclear ubiquitin E3 ligase involved in the degradation of chromatin-bound p65 and feedback termination of NF-κB transcriptional responses is PDZ and LIM domain 2 (PDLIM2). PDLIM2 is constitutively expressed in the nucleus and promotes the K48-linked polyubiquitylation of chromatin-bound p65 following TLR stimulation through its LIM domain [260]. The PDZ domain of PDLIM2 targets p65 to promyelocytic leukemia (PML) nuclear bodies, where it undergoes proteasomal degradation. Consequently, PDLIM2 deficiency leads to enhanced LPS-induced accumulation of nuclear p65, increased NF-κB-dependent gene expression and greater sensitivity to LPS-induced septic shock. Recently, the chaperone protein heat shock protein of 70 kD (HSP70) and the ubiquitin E3 ligase Makorin Ring Finger Protein 2 (MKRN2) have been shown to cooperate with PDLIM2 in the this feedback mechanism [260,261]. LPS stimulation triggers the nuclear translocation of HSP70 where it interacts with PDLIM2 and BCL2-associated athanogene 1 (BAG1) [262]. BAG1 and HSP70 cooperatively promote the transport of the ubiquitinated-p65:PDLIM2 complex to the proteasome to facilitate p65 degradation. MKRN2 cooperates with PDLIM2 to promote the polyubiquitylation and degradation of p65 in response to NF-κB-activating stimuli [261].

An example of promoter displacement as a mechanism of termination of NF-κB transcriptional responses is provided by the IκB proteins, which ‘strip’ NF-κB subunits from their cognate κB sites. Another example is provided by the SUMO E3 ligase, PIAS1, which also provides another example of a negative feedback mechanism directly dependent on IKK kinase activity, rather than *de novo* NF-κB-dependent expression [263]. PIAS1 is involved in the selective down-regulation of a subset of NF-κB dependent genes, particularly proinflammatory cytokines and chemokines [264]. A fraction of cellular IKKα is localised to the nucleus under basal conditions where it associates with PIAS1. Various NF-κB activating stimuli, including TNFα, IL-1 and LPS, promote the rapid IKKα-dependent phosphorylation of PIAS1 at S90 to trigger dissociation of PIAS1 from IKKα [263]. Newly dissociated PIAS1 rapidly displaces p65 from the promoters of specific genes to terminate their expression. Consistent with these findings, PIAS1-deficient mice are hypersenstitive to endotoxic shock [265]. Another member of the PIAS protein family, PIASy, may cooperate with PIAS1 in the termination of NF-κB signalling [266]. Indeed, PIASy-deficient mice are also hypersensitive to LPS-induced septic shock and exhibit enhanced expression of specific p65 target genes. PIASy may also block TRIF-dependent NF-κB activation [267]. A third PIAS family member, PIAS3, has also been implicated in the feedback regulation of p65 transcriptional activity. PIAS3 catalyses the SUMOylation of promoter-bound p65 in response to TNFα-dependent NF-κB activation to block p65-dependent transcription [268]. The mechanism of p65 SUMOylation-mediated transcriptional repression is unclear, but may involve interference with the recruitment of transcriptional co-activators, such as CREB-binding protein (CBP) [269], or the recruitment of transcriptional repressors. PIAS3 has also been proposed to negatively regulate NF-κB signalling through the inhibitory SUMOylation of TAB2 [270].

A final example is the transcriptional cofactor, CBP/p300 interacting transactivator with Glu/Asp rich carboxy-terminal domain 2 (CITED2). In macrophages CITED2 expression is induced by LPS stimulation in an NF-κB-dependent manner [271]. Newly synthesised CITED2 localises to the nucleus where it interacts with the NF-κB coactivator p300, preventing it's binding to p65 and thus impairing p65 acetylation, inhibiting retention of p65 at target promoters and inhibiting gene expression (Figure 14B).

## Post-transcriptional mechanisms of feedback inhibition

A more recently discovered class of negative feedback regulators are those NF-κB target gene products that act at the post-transcriptional level to modulate the stability and/or translation of specific mRNA transcripts encoding core NF-κB components. These include RNA-binding proteins (RBPs) that promote the decay of their mRNA targets and a growing list of microRNAs (miRNAs) that negatively regulate gene expression by targeting their mRNA targets for cleavage and/or translational silencing. A detailed description of the roles of miRNAs in regulating inflammation is beyond the scope of this review, and the interested reader is referred to the following excellent reviews on this topic [272,273]. The discussion here is limited to the handful of miRNA genes to which negative feedback functions in NF-κB signalling have been attributed.

### miRNAs as feedback regulators of NF-κB signalling

miR-146a was identified from a screen of 200 miRNAs for LPS-induced expression in THP-1 cells [274], is an NF-κB target gene and serves as a negative feedback regulator by targeting TRAF6 and IRAK1 for post-transcriptional repression, reducing NF-κB activation. miR-146a-deficient mice exhibit severe immune defects, including hypersensitivity to LPS-induced shock, myeloproliferation and spontaneous autoimmune disorder due to loss of peripheral T cell tolerance [275]. miR-146a KO mice also develop secondary lymphoid tumours suggesting a tumour suppressor function for miR-146a. Indeed, miR-146a expression is down-regulated in lymphoma [276], gastric [277], cervical [278] and thyroid cancers [279].

Another example of a miRNA negative feedback regulator of NF-κB signalling is miR-21, which is induced by LPS stimulation in mouse and human macrophages in an NF-κB-dependent manner [280]. miR-21 attenuates TLR4-induced NF-κB activity by targeting programmed cell death protein 4 (PDCD4) for post-transcriptional repression, thereby reducing expression of pro-inflammatory NF-κB target genes, including *IL-6*. The mechanism by which PDCD4 promotes NF-κB signalling is currently unclear but appears to be indirect and secondary to its function as a translational repressor. miR-21 expression is also up-regulated during liver regeneration to inhibit NF-κB activation through suppression of *PELLINO-1* expression [281].

miR-9 expression is induced by diverse MyD88-activating TLR agonists, including LPS, and proinflammatory cytokines, including TNFα and IL-1β in an NF-κB-dependent manner and acts as a feedback regulator of NF-κB signalling in human monocytes and neutrophils [282]. The *NFKB1* gene (encoding p105) is a molecular target of miR-9; p50, the processed form of p105, forms heterodimers with other NF-κB subunits [283] but due to their lack of TADs p50 homodimers act as transcriptional repressors with anti-inflammatory roles in the termination of transcription of proinflammatory cytokines [284]. Overexpression of p50 homodimers in tumour-associated macrophages promotes a state of proinflammatory tolerance that inhibits M1 antitumour responses [285] so the induction of miR-9 may act as a fine-tuning mechanism to restrict the unbalanced expression of anti-inflammatory p50 homodimers.

miR-302b is a negative feedback regulator of NF-κB signalling during the host immune response to bacterial infection [286]. miR-302b expression is induced following bacterial infection in a manner dependent on TLR2/TLR4-mediated NF-κB, ERK and p38 signalling. In turn, miR-302b suppresses bacterium-induced pro-inflammatory NF-κB signalling by targeting IRAK4 for post-transcriptional repression. miR-147 is also strongly up-regulated in LPS-treated macrophages in a manner dependent on both MyD88 and TRIF pathways, and hence NF-κB and IRF3, respectively [287]. miR-147 knockdown enhances, while miR-147 mimics attenuate TLR2/3/4-stimulation induced inflammatory cytokine expression. However, a molecular target of miR-147 within the TLR signalling pathway has yet to be identified.

Finally, miR-718 is another negative feedback regulator of TLR-induced NF-κB signalling [288]. LPS stimulation induces the expression of miR-718 in murine macrophages although the NF-κB-dependency of this expression awaits definition. miR-718 targets PTEN mRNA for degradation, leading to enhanced PI3 K/AKT signalling which correlates with decreased expression of proinflammatory cytokines. Indeed, the AKT pathway has been proposed to suppress NF-κB activation and the expression of proinflammatory cytokines in the early phase of the innate immune response in monocytes/macrophages [289,290]. Activated AKT is also proposed to indirectly down-regulate NF-κB signalling through the induction of the miRNA, let-7e, which subsequently down-regulates *TLR4* to further inhibit TLR4-mediated NF-κB activation [291].

### RNA binding proteins as feedback regulators of NF-κB signalling

The mRNA transcripts of various NF-κB signalling components and proinflammatory mediators are also targeted for post-transcriptional repression by the actions of a group of CCCH zinc finger RBPs: tristetraprolin (TTP), regulatory RNase-1 (Regnase-1) and Roquin-1. These proteins coordinate a regulatory network that maintains immune homeostasis, controls adaptive immune responses and promotes the timely resolution of inflammation by modulating signalling pathways, including NF-κB, primarily via mRNA decay. TTP and Regnase-1 are induced in an NF-κB-dependent manner and act as negative feedback regulators of NF-κB signalling. For example, TTP, encoded by the *ZFP36* gene of the Tis11-family, is strongly induced by proinflammatory stimuli, including LPS and TNFα, in an NF-κB-dependent manner [292]. TTP binds to AU-rich elements (AREs) in the 3′UTRs of specific mRNAs, including those encoding the cytokines TNFα, IL-6 and IL-23, and mediates the recruitment of factors that inhibit the translation of the target mRNA and increase the rate of decay. In the case of TNFα mRNA, TTP recruits the CCR4-CAF1-NOT1 deadenylase complex, the DCP2 decapping complex and the 4EHP-GYF2 cap-binding complex to promote TNFα mRNA decay and translation inhibition, respectively [293–295]. Consequently, TTP-deficient mice exhibit drastically increased sensitivity to LPS-induced septic shock and develop cachexia, dermatitis, arthritis and autoimmunity, most of which can be rescued by treatment with TNF-blocking antibodies or crossing with TNFR1-deficient mice [296].

In the early stage of the NF-κB response TTP activity is inhibited through p38-MK2-dependent phosphorylation, enabling the translation of its target mRNAs [297]. At later stages (>4 h LPS treatment), TTP phosphorylation diminishes and TTP expression increases, leading to enhanced mRNA decay and feedback inhibition of NF-κB signalling. TTP also has inhibitory functions within the NF-κB pathway that are independent of its RNA-binding activity. For example, TTP directly interacts with the p65 subunit to interfere with its nuclear import [298] and modulates recruitment of transcriptional corepressors/coactivators, such as HDAC1 and CBP, to chromatin-bound p65 to suppress NF-κB transcriptional activity [299].

Regnase-1, encoded by the *ZC3H12A* gene, is a RNA-binding protein induced by LPS, IL-1β, TNFα and genotoxic stress in an NF-κB-dependent manner [300–302] and with roles in the negative feedback inhibition of NF-κB signalling. Similar to TTP, Regnase-1 activity is temporally regulated at the transcriptional, post-transcriptional and post-translational level during the course of the inflammatory response. In the early phase of LPS- and IL-1β- (but not TNFα-) induced macrophage activation Regnase-1 undergoes rapid IKK-dependent phosphorylation, targeting it for β-TrCP-mediated proteasomal degradation and providing a window during which Regnase-1 targets are expressed [303]. Similarly, in the early phase of T cell activation Regnase-1 undergoes TCR-induced MALT1-dependent cleavage [304]. Later in the response (>240 min after macrophage stimulation), Regnase-1 mRNA is reexpressed to promote the decay of its proinflammatory mRNA targets.

In cooperation with the helicase UPF1, Regnase-1 promotes degradation of a subset of stem–loop-containing mRNAs via its intrinsic endonuclease activity [305,306]. Regnase-1 controls the early phase of inflammation by targeting a subset of translationally active mRNAs including IL-6 and IL-1β in macrophages and Icos, c-Rel and Ox40 in T cells [306]. However, the role of Regnase-1 in feedback inhibition of NF-κB signalling has been questioned by the lack of LPS-induced NF-κB hyperactivation in macrophages from Regnase-1-deficient mice [305], although this may reflect redundancy with TTP and/or Roquin, which target overlapping sets of mRNAs. Regnase-1-deficient mice do develop systemic inflammatory syndrome and are highly sensitive to LPS-induced septic shock due to hyperactive TNFα synthesis. Inhibition of c-Rel expression is important for negative regulation of T cell hyperactivation by Regnase-1 [304]. Regnase-1 may also mediate negative feedback control of NF-κB signalling in a manner independent of its RNase activity. Regnase-1 is proposed act as an adaptor molecule that recruits TANK and USP10 to form a complex that inhibits NF-κB induced by genotoxic stress and/or IL-1β/LPS stimulation by promoting the deubiquitylation of NEMO and TRAF6 [137,302].

Finally, Roquin-1, encoded by the *RC3H1* gene, is a RBP that recognises a conserved stem–loop motif, called the constitutive decay element (CDE) in the 3'UTR of its target mRNAs through interactions with its CCCH-type ZnF and ROQ domains [307]. Roquin-1 and Regnase-1 regulate an overlapping set of mRNAs, including IL-6, due to the recognition of a common stem–loop structure. However, Roquin-1 controls the later phases of inflammation by degrading translationally inactive mRNAs [306]. In addition, Roquin-1 can enhance NF-κB activation by promoting decay of A20 mRNA [308].

## Deregulation of NF-κB negative feedback controls in human disease

Canonical NF-κB signalling plays essential roles in immune system development, host defence and tissue homeostasis through the regulation of genes involved in cell proliferation, survival, immune responses, inflammation, adhesion, metabolism, angiogenesis and differentiation. However, the enormous redundancy of negative feedback mechanisms outlined above emphasises the importance of appropriate temporal control and termination of NF-κB signalling for the maintenance of tissue homeostasis. Indeed, aberrant NF-κB activity is a common factor in the development of many chronic autoimmune, inflammatory, degenerative and malignant diseases and loss of function of negative feedback regulators of NF-κB signalling and subsequent aberrant NF-κB activation is commonly associated with human disease. Here we highlight a few illustrative examples.

A number of negative feedback regulators of NF-κB signalling have been identified through linkage analysis and more recently genome wide association studies (GWAS) as susceptibility loci for various inflammatory and autoimmune diseases. For example, variants (including SNPs and mutations in the coding region) of the negative feedback regulator of TLR/IL-1R signalling, IRAK-M are associated with early-onset persistent asthma [309]. Germline SNPs in *TNFAIP3* (encoding A20) are associated with Crohn's disease [310], coeliac disease [311], type 1 diabetes [312], RA [313,314], coronary artery disease in type 2 diabetes [315], systemic sclerosis [316], psoriasis [317] and systemic lupus erythematosus (SLE) [318,319]. In multiple cases these disease-associated SNPs have been shown to reduce A20 function or expression. For example, a SNP in the 3′ non-coding region confers susceptibility to SLE by reducing A20 mRNA and protein expression [320]. SNPs in *TNIP1* (encoding ABIN-1, a component of the A20 ubiquitin-editing complex) are also strongly linked with susceptibility to SLE and psoriatic arthritis [317]. Germline frameshift mutations resulting in A20 haploinsufficency have also been identified in patients with an autoimmune lymphoproliferative syndrome-like phenotype (ALPS) and T cells from these patients exhibit increased NF-κB activation [321]. Germline loss-of-function mutations in *TNFAIP3* leading to A20 haploinsufficency have also been shown to cause Behcet-like autoimmunity, which is characterised by mucosal ulcers and eye inflammation [322]. *CYLD* germline polymorphisms are associated with IBD [323], while germline mutations in *CYLD* are responsible for familial cylindromatosis; a disease characterised by benign tumours of the sweat glands around the head, face and neck [324]. Furthermore, germline SNPs in *TRAF1* are risk loci for RA [325–327]. Consistent with the phenotype of TRAF1-deficient mice, monocytes from patients with a common SNP (rs3761847) in *TRAF1* express lower TRAF1 protein and exhibited enhanced cytokine production in response to LPS challenge compared with cells from healthy patients [173]. IκBα promoter SNPs are associated with susceptibility to Sjögren's syndrome (SS) in humans, an autoimmune disease characterised by inflammatory infiltration of lymphocytes into lung, salivary, and lacrimal glands [328]. Patients also exhibit an increased risk of developing non-Hodgkin lymphoma. In support of this disease association, mutation of κB enhancer sites in the IκBα promoter in mice leads to development of SS, abnormal T cell development and hypersensitivity to septic shock, owing to a defective IκBα feedback loop, leading to constitutive NF-κB activation and deregulated expression of inflammatory mediators [329]. Lower IκBα expression has also been confirmed to promote NF-κB activation in monocytes from primary SS patients [330].

Human genetic studies have also linked somatic mutations of negative feedback regulators of NF-κB signalling with inflammatory and malignant human diseases. For example, A20 is commonly inactivated by biallelic somatic loss of function mutations or genomic deletions in various B cell lymphomas, including marginal zone lymphomas, diffuse large B-cell lymphoma (DLBCL), MALT lymphoma, primary mediastinal B cell lymphomas and Hodgkin's lymphoma [331–334]. A20 is also targeted for constitutive proteolytic cleavage by MALT1, which drives constitutive NF-κB activation in activated B-cell (ABC) DLBCL [335]. The expression of *TNFAIP3* may also be inhibited by methylation of its promoter [336]. A20-mediated restriction of NF-κB signalling is strongly linked to the tumour suppressive function of A20 in B cells. For example, A20 reexpression in lymphoma-derived cell lines lacking functional *TNFAIP3* alleles induces cell cycle arrest/cell death with concomitant down-regulation of constitutive NF-κB activation [331]. A20 is also a putative tumour suppressor in Sézary syndrome, a T-cell malignancy [337]. Somatic mutations in *TNIP1* (encoding ABIN-1) have also been found in various human lymphomas [338], while somatic mutations in *NFKBIA* (encoding IκBα) have been observed in Hodgkin's disease (Jungnickel et al. 2000) and in glioblastomas [339].

Aberrantly expressed miRNAs targeting negative feedback regulators of NF-κB signalling may also act as potential drivers of tumourigenesis and autoimmune disease (see Table 1). A representative example is provided by miR-486, which is substantially overexpressed in human gliomas and whose expression strongly correlates with glioma tumour grade and inversely with patient survival [340]. mir-486 targets CYLD and Cezanne and multiple A20-interaction partners, including ITCH, ABIN-1, ABIN-2 and ABIN-3, leading to constitutive and enhanced/prolonged signal-induced NF-κB activation, which promotes aggressiveness and malignancy in patient-derived glioma cells [340]. Other examples are highlighted in Table 1 together with their gene targets and disease association.

**Table 1:** miRNAs targeting negative feedback regulators of NF-kB signalling in human disease

miRNA	Targets	Disease type	Reference
miR-486	CYLD, Cezanne, ITCH, ABIN-1, ABIN-2, ABIN-3	Glioblastoma	Song et al. [340]
miR-500	TAX1BP1, Cezanne, CYLD	Gastric cancer	Zhang et al. [341]
miR-1180	Cezanne, ABIN-2	Hepatocellular carcinoma	Tan et al. [342]
Mir-19	CYLD	T-cell acute lymphoblastic leukemia	Ye et al. [343]
mir-19b	A20, RNF11, SOCS-1	Rheumatoid arthritis	Gantier et al. [344]
mir-181b	CYLD	Breast, colon cancer etc	Iliopoulos et al. [345]
mir-30e*	IκBα	Glioblastoma	Jiang et al. [346]
miR-182	CYLD	Glioblastoma	Song et al. [347]
mir-125b	A20	Glioblastoma	Haemmig et al. [348]
mir-17∼92	CYLD, A20, RNF11, TAX1BP1, TRAF3	Lymphomas	Jin et al. [349]
mir-196a	IκBα	Pancreatic cancer	Huang et al. [350]
mir-362	CYLD	Gastric cancer	Xia et al. [351]
mir-372	TNFAIP1	Gastric cancer	Zhou et al. [352]

Although the main focus of this review is the negative feedback control of NF-κB, it would be remiss not to mention positive feedback mechanisms that also lead to human disease. Not only does TNFα initiate the canonical NF-κB pathway, but TNFα is itself a NF-κB target gene, therefore a positive feedback loop is created until the signals perpetuating this cascade are eliminated; this is also the case for IL-1. Furthermore, this positive feedback loop may be reinforced by continued binding of an activating ligand to its receptor. This autocrine and paracrine regulation was first observed in ovarian cancer where TNFα acts as a growth factor [353]. Aberrant increased activity of NF-κB as induced by TNFα is detected in patients with autoimmune and chronic inflammatory diseases such as SLE and RA, further highlighting the possible catastrophic consequences of this positive feedback loop [354]. Additionally, this loop has also been implicated in promoting acute myeloid leukaemia (AML) [355]. Another powerful feed-forward mechanism involves the p65 subunit of NF-κB driving its own transcription [356]. One study found that this loop is perpetuated by high doses of LPS and becomes dominant over negative feedback loops. Modelling and live-cell imaging suggested that in immune cells there is a switch in the relative dominance of positive and negative feedback loops which may explain how they balance opposing signals and how they set a response threshold for the host [357].

## Conclusions

Feedback regulation of the signalling pathways leading to NF-κB activation is clearly very important to ensure that the magnitude and duration of cellular responses are appropriate; this is perhaps best evidenced by the disease phenotypes associated with genetic defects in feedback modulators such as A20, CYLD and IκBα. However, it is important to recognise that the influence of feedback controls is not simply on the magnitude and duration NF-κB activation; it may also influence coincident signalling arising from differential effects on multiple, parallel pathways. NF-κB-dependent feedback is complex and acts at multiple levels from individual receptors, through signalosome complexes, the IKKs themselves down to individual NF-κB subunits in the nucleus. Critically, other signalling pathways parallel to the core IKK-NF-κB pathway are activated at certain steps down these pathways and their activation may or may not be influenced by NF-κB-dependent feedback modulators. For example, RIPK1 in concert with FADD can drive activation of caspase-8 and apoptosis. In addition, TAK1 not only activates the IKK complex during canonical TNFα signalling; it also activates MAP kinase kinases that lead to activation of the JNK and p38 stress kinase pathways and AP-1-dependent gene expression. So whilst NF-κB-dependent feedback modulators that act at the receptor level (such as decoy receptors) will shut down all signalling, feedback modulators that act at or below the IKK complex may exert more selective effects on NF-κB activation, leaving JNK and p38 signalling intact. These qualitative changes in signalling and gene expression will alter the signalling response to TNFα, and other stumuli, thereby influencing cell fate.

As we understand more about these signalling pathways and are able to monitor NF-κB activation in live single cells we are also learning more about how these feedback loops shape the responses of individual cells. For example, IκB-mediated oscillations in nuclear NF-κB are a normal and important characteristic of the response of NF-κB to TNFα and likely other stimuli. Variation in NF-κB oscillation frequencies, including by different IκBs, alters gene-expression profiles suggesting that oscillatory dynamics can exert qualitative effects on gene expression, including inflammatory cytokine responses [236]. They will also likely contribute to cellular heterogeneity within a population of cells, perhaps providing an explanation for how NFκB can regulate diverse cellular outcomes, including cell survival or death and or division or differentiation.

Negative feedback loops also confer robustness and control over signalling pathways. They allow pathways to adapt to specific interventions and while essential for the normal functioning of cells, tissues and organisms exposed to stimuli, they may represent a challenge for the development of drugs that target signalling pathways. This is seen most obviously with the negative feedback loops that operate in RAS-RAF-MEK-ERK1/2 signalling, which give the pathway the properties of a negative feedback amplifier [358]. For example, ERK1/2 feedback phosphorylation of RAF proteins impairs RAS-dependent RAF dimerisation, which is critical for activation of wild type RAF proteins and downstream signalling [359]. Consequently, whilst inhibitors of MEK or ERK1/2 will disrupt downstream signalling, they will also prevent ERK1/2-mediated feedback phosphorylation of RAF, resulting in further RAF activation; this drives the rapid ‘rebound’ reactivation of ERK1/2 signalling that is observed in RAS-mutant tumour cells with wild type RAF proteins. The use of intra-pathway drug combinations (for example, a RAF inhibitor plus a MEK inhibitor) can mitigate pathway reactivation arising from loss of feedback inhibition [360]. However, class 1 BRAF mutants (e.g. the most common BRAF mutation, BRAF^V600E^) signal constitutively as monomers, independent of RAS, so they are not susceptible to ERK1/2-mediated feedback phosphorylation. Thus, a disease-driving BRAF^V600E^ mutation is oncogenic both because it exhibits constitutive activity and because it is refractory to pathway limiting feedback phosphorylation. Another example is the relative insensitivity of BRAF^V600E^ colorectal cancer cells to selective BRAF inhibitors [361] due to loss ERK1/2-mediated feedback inhibition of EGFR activity; treatment of these cells with BRAF inhibitors (or MEK inhibitors) in combination EGFR inhibitors is synergistic because it prevents the activation of EGFR that occurs following ERK1/2 inhibition. Thus liberation from ERK1/2-mediated feedback phosphorylation may contribute to the oncogenic properties of mutant RAF proteins or wild type EGFR and their response to very selective clinically approved inhibitors.

To what extent lessons can be learnt from the experiences with RAF, MEK and ERK inhibitors and feedback and applied to NF-κB signalling is currently unclear. The main reason for this is that drug discovery efforts are far less advanced for the NF-κB signalling pathway than they are for the RAF-MEK-ERK1/2 pathway. The rapid advance in our understanding of the role of feedback in responses to RAF, MEK or ERK inhibitors reflects the fact that these agents are all highly selective (even ‘specific’ in the case of allosteric MEK inhibitors) and extremely potent (typically biologically active in the 1–100 nM dose range). This is critical as it means we can have confidence that the effects of the drugs on feedback loops are very likely to reflect ‘on-target’ activity. Sadly, the same cannot currently be said for inhibitors of NF-κB signalling. With regards to the IKK complex, efforts to date have focused largely on searching for inhibitors of IKKβ; however, most have failed in the clinic due to poor potency and/or selectivity. Probably the best IKKβ inhibitors for research purposes at the current time are BI605906 and MLN-120B, which exhibit 300-fold and 50-fold selectivity for IKKβ over IKKα [362]. The development of potent selective IKKα inhibitors has lagged behind that for IKKβ, but suitable tool compounds have now been reported [363] and may find utility in prostate cancer [364]. Progress is also now being made upstream of the core IKK complex with the development of potent and selective inhibitors of TAK1 and NIK. Takinib is a potent (9 nM) and highly selective inhibitor of TAK1 [365] that exhibits activity in arthritis models [366] and in a Hodgkin's Lymphoma model with mutation of the A20 DUB [367]. In addition, potent and selective inhibitors of NIK are now becoming available [368] including XT2, which is potent (9 nM) and effective at inhibiting NIK-induced inflammatory signalling [369]. With these tools we now have potent and selective agents that we can use with increased confidence to interrogate the effects of NF-κB pathway inhibition on feedback loops and pathway rebound. For example, will the feedback phosphorylation and inhibition of NIK by IKKα [370] influence the efficacy of IKKα inhibitors by stabilising NIK which will then activate additional pools of IKKα? Other scenarios can certainly be envisaged and this should be anticipated as these more selective inhibitors of NF-κB signalling progress.

## Abbreviations

ABIN1: A20-binding inhibitor of NF-κB
AhR: Aryl hydrocarbon receptor
AML: Acute Myeloid Leukaemia
ANK: Ankyrin
AP-1: Activator protein 1
ARD: Ankyrin repeat domain
ARE: AU-rich element
ATM: Ataxia telangiectasia mutated
ATP: Adenosine triphosphate
AUEC: A20 ubiquitin-editing complex
BAFF: B-cell activating factor
BAG1: BCL2-associated athanogene 1
BCL-2/3: B-cell lymphoma 2/3
BCR: B cell receptor
BMDM: Bone marrow derived macrophage
BRAF: v-raf murine sarcoma viral oncogene homolog B1
CAC: Colitis-associated cancer
CARD: Caspase activation and recruitment domain
CBP: CREB-binding protein
CCR: C chemokine receptor
CD: Cluster of differentiation
CDE: Constitutive decay element
cIAP1/2: Cellular inhibitor of protein 1/2
CITED2: CBP/p300 interacting transactivator with Glu/Asp rich carboxy-terminal domain 2
COMMD1: Copper metabolism mouse U2af1-rs1 region 1-domain-containing protein 1
CoREST: Corepressor to RE1 silencing transcription factor
COX2: Cytochrome c oxidase 2
CSC: Cancer stem cell
CXCL: Chemokine CXC motif ligand
CYLD: Cylindromatosis
DAMP: Damage associate molecular patterns
DLBCL: Diffuse large B-cell lymphoma
DUB: Deubiquitinase
ERK: Extracellular signal-regulated kinase
FADD: Fas-associated protein with death domain
G-CSF: Granulocyte-colony stimulating factor
GRR: Glycine rich region
GSK3β: Glycogen synthase kinase 3 beta
HCC: Hepatocellular carcinoma
HDAC-1: Histone deacetylase 1
HECT: Homologous to the E6-AP carboxyl terminus
HSP: Heat shock protein
IBD: Inflammatory bowel disease
IER: Immediate early response
IFN: Interferon
IKK: IκB kinase
IL: Interleukin
IL-1R: Interleukin-1 receptor
IRAK: Interleukin-1 receptor-associated kinase
IRF: Interferon regulatory transcription factor
IκB: Inhibitor of κB
JAMM: JAB1/MPN/Mov34 metalloenzyme domain
JNK: c-Jun N-terminal kinase
kDa: Kilodalton
LPS: Lipopolysaccharide
LSD1: Lysine-specific demethylase 1
LTβR: Lymphotoxin β receptor
LUBAC: Linear ubiquitin chain assembly complex
LZ: Leucine zipper
MAL: MyD88 adaptor-like
MALT1: Mucosa-associated lymphoid tissue lymphoma translocation 1
MAPK: Mitogen activated protein kinase
MD-2: Myeloid differentiation factor 2
MEF: Mouse embryonic fibroblast
MEK: MAPK/ERK Kinase
MINDY: Motif interacting with ubiquitin-containing novel DUB
MKRN2: Makorin ring finger protein 2
MyD88: Myeloid differentiation primary response protein 88
NEMO: NF-κB essential modulator
NES: Nuclear export signal
NF-κB: Nuclear factor-kappa B
NIK: NF-κB-inducing kinase
NKILA: NF-κB interacting long non-coding RNA
NLR: Nod-like receptor
NLRC: NLR family card domain
NLS: Nuclear localisation signal
N-terminal: Amino terminal
Nurr1: Nuclear receptor related protein 1
OPTN: Optineurin
OTU: Ovarian tumour
PAMP: Pathogen-associated molecular patterns
PDCD4: Programmed cell death protein 4
PDLIM2: PDZ and LIM domain 2
PEST: Region rich in proline, glutamate, serine and threonine residues
PIAS: Protein inhibitor of STAT
PTM: Post-translational modification
RA: Rheumatoid arthritis
RANK: Receptor activation of NF-κB
RBP: RNA-binding protein
RHD: Rel homology domain
RING: Really interesting new gene
RIPK1: Receptor interacting serine/threonine protein kinase 1
RP105: Radioactive protein 105 kDa
SC: Signalosome complex
SCF: Skp cullin F-box-containing complex
SENP: Sentrin/SUMO-specific protease
SIGIRR: Single Ig IL-1 receptor-related molecule
SLE: Systemic lupus erythematosus
SMRT: Silencing mediator for Retinoid/Thyroid hormone receptors
SNP: Single nucleotide polymorphism
SOCS1/3: Suppressor of cytokine signalling 1/3
SPATA2: Spermatogenesis associated protein 2
SS: Sjögren's syndrome
sST2: Serum stimulation 2
TAB: TAK1-binding protein
TAD: Transactivation domain
TAK: Transforming growth factor beta (TGFβ)-activated kinase
TCR: T cell receptor
Th: T helper cell
TIR: Toll/interleukin-1 receptor
TIRAP: TIR-domain-containing adapter protein
TLR: Toll-like receptor
TNF: Tumour necrosis factor
TNFR: Tumour necrosis factor receptor
TRADD: TNF-receptor type 1-associated death domain protein
TRAF: Tumour necrosis factor receptor-associated factor
TRAM: TRIF-related adaptor molecule
TRIB3: Tribbles homolog 3
TRIF: TIR-domain-containing adaptor protein inducing IFNβ
TTP: Tristetraprolin
Ub: Ubiquitin
UBC: Ubiquitin-conjugating enzyme
UBD: Ubiquitin binding domain
UC: Ulcerative colitis
UCH: Ubiquitin C-terminal hydrolase
USP: Ubiquitin specific protease
WIP1: WT p52-induced phosphatase 1
WT: Wild-type
XIAP: X-linked inhibitor of apoptosis protein
βTrCP: Beta-transducin repeat containing protein
